# Mechanistic Insight for Disinfection Byproduct Formation Potential of Peracetic Acid and Performic Acid in Halide-Containing Water

**DOI:** 10.1021/acs.est.3c00670

**Published:** 2023-07-25

**Authors:** Junyue Wang, Jiale Xu, Juhee Kim, Ching-Hua Huang

**Affiliations:** School of Civil and Environmental Engineering, Georgia Institute of Technology, Atlanta, Georgia 30332, United States

**Keywords:** peroxyacids, peracetic acid, performic acid, halogenated disinfection byproducts, saline wastewater, kinetic modeling

## Abstract

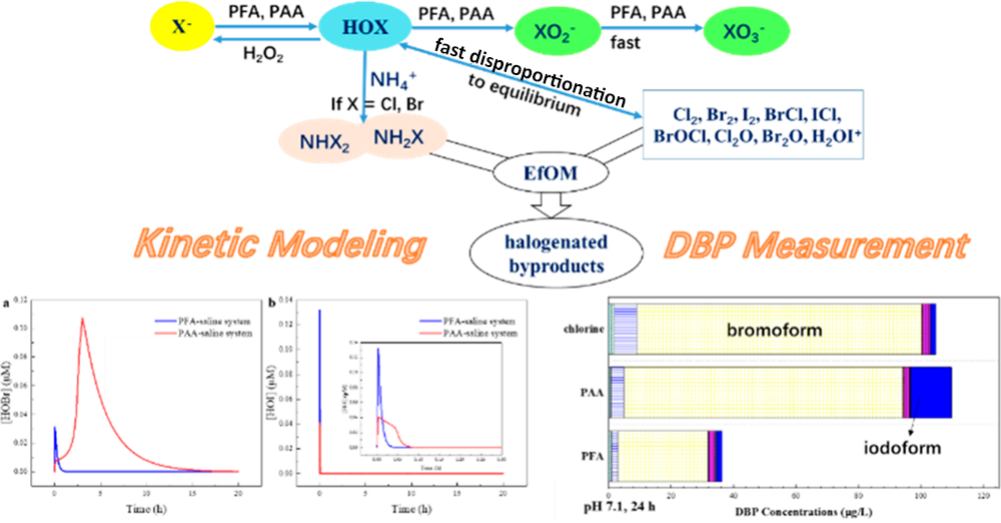

Peracetic acid (PAA) and performic acid (PFA) are two major peroxyacid (POA) oxidants of growing usage. This study reports the first systematic evaluation of PAA, PFA, and chlorine for their disinfection byproduct (DBP) formation potential in wastewater with or without high halide (i.e., bromide or iodide) concentrations. Compared with chlorine, DBP formation by PAA and PFA was minimal in regular wastewater. However, during 24 h disinfection of saline wastewater, PAA surprisingly produced more brominated and iodinated DBPs than chlorine, while PFA effectively kept all tested DBPs at bay. To understand these phenomena, a kinetic model was developed based on the literature and an additional kinetic investigation of POA decay and DBP (e.g., bromate, iodate, and iodophenol) generation in the POA/halide systems. The results show that PFA not only oxidizes halides 4–5 times faster than PAA to the corresponding HOBr or HOI but also efficiently oxidizes HOI/IO^–^ to IO_3_^–^, thereby mitigating iodinated DBP formation. Additionally, PFA’s rapid self-decay and slow release of H_2_O_2_ limit the HOBr level over the long-term oxidation in bromide-containing water. For saline water, this paper reveals the DBP formation potential of PAA and identifies PFA as an alternative to minimize DBPs. The new kinetic model is useful to optimize oxidant selection and elucidate involved DBP chemistry.

## Abstract 2

This paper reveals the disinfection byproduct formation potential of peracetic acid in saline wastewater and identifies performic acid as an alternative, through evaluation of experimental data with a comprehensive kinetic model.

## Introduction

Disinfection byproducts (DBPs) are organic compounds generated from the interaction between oxidants and organic matter or halides, during food, water, and wastewater disinfection.^1−4^ Thus far, more than 700 toxic, mutagenic, or carcinogenic DBPs have been identified in drinking water,^2,5−7^ wastewater effluents,^8,9^ swimming pools,^10^ and food processing facilities,^3,11^ leading to chronic public health problems and ecological risks in aquatic ecosystems.^1,4,12,13^

Free chlorine (HOCl/ClO^–^), monochloramine (NH_2_Cl), ozone (O_3_), and UV irradiation are among the most widely applied disinfectants, and their capacity for DBP formation is discussed briefly as follows. Free chlorine produces DBPs in three major pathways: (1) oxidizing bulk aromatic organic matter to smaller carbonyl^14−16^ or phenolic compounds,^17^ with further conversion to carbonaceous DBPs (C-DBPs), including trihalomethanes (THMs), haloacetic acids (HAAs), haloacetaldehydes (HALs), and chlorinated aromatic compounds,^18−21^ (2) oxidizing amines to nitrogenous DBPs (N-DBPs), such as halonitromethanes (HNMs) and haloacetonitriles (HANs) (could be further hydrolyzed to haloacetamides (HAMs)),^22,23^ and (3) when applied as breakpoint chlorination in ammoniacal water, producing dichloramine and reactive nitrogen species (e.g., HNO, ONOOH), which in turn generates nitro(so) DBPs.^24,25^ In contrast, monochloramine is mainly responsible for N-DBP formation, owing to its reactions with amines^26,27^ and aldehydes^28^ to produce *N*-nitrosamines and HANs, respectively. Ozone leads to the formation of nitromethanes^29−31^ and bromate.^32^ UV irradiation may produce reactive nitrogen species (e.g., ^•^NO, ^•^NO_2_) in the presence of nitrite, nitrate, or chloramines, hence enhancing the formation of nitro(so) DBPs.^33−35^

Recently, peroxyacids (POAs) (i.e., peracetic acid (CH_3_C(O)OOH, PAA) and performic acid (HC(O)OOH, PFA)) have been proposed as an alternative to the aforementioned traditional disinfectants.^9,11,36−38^ PAA has a p*K*_a_ of 8.2, and its bacterial inactivation capacity is comparable to that of chlorine.^39,40^ PFA has a p*K*_a_ of 7.3;^41^ its bacterial inactivation efficacy usually outperforms PAA, despite its faster self-decay.^38,42^ POAs do not contain halogen elements, and the coexistent hydrogen peroxide (H_2_O_2_) in the POA solution can reduce halogenating agents in the water matrix.^43^ Furthermore, PAA’s sluggish reactivity toward amine compounds limits the potential of N-DBP formation.^40,44^ Nevertheless, in halide-containing water, PAA could oxidize bromide (Br^–^) and iodide (I^–^) to free bromine (HOBr/BrO^–^) and free iodine (HOI/IO^–^), respectively, which in turn produces Br- and I-DBPs. In some previous studies, PAA indeed generated more dibromoacetic acid^45^ and iodinated THMs (I-THMs)^36^ than chlorine in halide-containing waters; hence, the capacity of PAA on DBP control deserves further investigation. Moreover, a literature search indicates that the DBP formation potential of PFA has never been studied.

As mentioned above, the major DBP formation pathway of PAA and PFA is their oxidation of halides, particularly Br^–^/I^–^, into halogenating agents (i.e., HOBr and HOI). Moreover, POA may further oxidize HOBr and HOI, which in turn shifts the byproducts from organic Br- and I-DBPs to bromate (BrO_3_^–^, toxic) and iodate (IO_3_^–^, nontoxic), respectively.^46−48^ Thus, to understand the DBP production by PAA and PFA, their reactivity with halides (particularly Br^–^, I^–^), HOBr/BrO^–^, and HOI/IO^–^ (rate constants denoted as *k*_Br^–^_, *k*_I^–^_, *k*_BrO^–^_, and *k*_IO^–^_ in Table 1) is of great importance. However, thus far, only the reaction rate constants between PAA and halides have been reported, while the reactions of PAA with HOBr/BrO^–^ and HOI/IO^–^ and the reactions involving PFA have not been studied (Table 1).^43^ These rate constants for other typical oxidants, including free chlorine,^47−49^ monochloramine,^50,51^ ferrate(VI),^52,53^ permanganate,^54^ and ozone^32,45^ have all been well-studied (Table 1).

**Table 1:** Apparent Second-Order Rate Constants between Oxidants and Br^–^, I^–^, BrO^–^, and IO^–^ (pH 7.0–7.2)

	*k*_Br^–^_	*k*_BrO^–^_	ref	*k*_I^–^_	*k*_IO^–^_	ref
HOCl/ClO^–^	1550	slow	(32)	4.3 × 10^8^	21	(67)
NH_2_Cl	1.4 × 10^–2^ a	2.7 × 10^5^ b	(50)	2.4 × 10^3^	<2 × 10^–3^	(67)
HOBr/BrO^–^		slow	(46)	5.0 × 10^9^	752	(47)
O_3_	160–258	1.58	(32)	2.0 × 10^9^	3.7 × 10^4^	(67)
KMnO_4_	unknown	unknown		7.0	6.9	(67)
ferrate(VI)	0.1–10.9	unknown	(32)	2.0 × 10^4^	2.0 × 10^3^	(67)
ClO_2_	<0.05	unknown	(46)	1900^c^	unknown	(67)
PMS	0.7	slow	(32)	1.1 × 10^3^	7.9 × 10^2^	(67)
PFA	1.03	slow	(43), this study	2.47 × 10^3^	120	this study
PAA	0.22	slow	(43), this study	3.89 × 10^2^	16	this study

a This reaction produces NH_2_Br and is reported at pH 7.5;^48^ other reactions for *k*_Br^–^_ all produce HOBr.

b This reaction produces NHBrCl and is reported at pH 7.5;^48^ other reactions for *k*_BrO^–^_ all produce BrO_2_^–^.

c The reaction produces I^•^;^49^ other reactions for *k*_I^–^_ all produce HOI.

Therefore, the objectives of this study were to (1) comprehensively compare the DBP formation by POAs [i.e., PAA and PFA in this study] and chlorine in regular and halide-containing wastewater; (2) determine the rate constants of POAs with halides, HOBr/BrO^–^, HOI/IO^–^, and other water matrix constituents [e.g., effluent organic matter (EfOM), NH_4_^+^]; and (3) establish a kinetic model that can predict the levels of HOBr and HOI during POA oxidation of halide-containing water.

## Materials and Methods

### Chemicals and Reagents

PAA solution, H_2_O_2_ solution, and free chlorine (NaOCl) solution were purchased from Sigma-Aldrich (St. Louis, MO). PFA (w/60% H_2_O_2_, molar ratio) was freshly synthesized following the method described in Text S1 in the Supporting Information.^38^ It should be noted that additional H_2_O_2_ was added to PAA working solutions in DBP formation experiments to reach a similar coexistent H_2_O_2_ concentration as PFA for a fair comparison. The additional H_2_O_2_ did not affect PAA concentration^40,55,56^ and was confirmed by experiments. Free bromine was freshly synthesized according to a method by Guo et al. by mixing sodium hypochlorite and bromide at a molar ratio of 1:1.05 and adjusted the pH to 11.0.^57^

Standard chemicals for DBPs, including THM4 (trichloromethane (TCM), dichlorobromomethane (DCBM), dibromochloromethane (DBCM), tribromomethane (TBM)), three HANs (dichloroacetonitrile (DCAN), bromochloroacetonitrile (BCAN), dibromoacetonitrile (DBAN), trichloroacetonitrile (TCAN)), one HNM (trichloronitromethane (TCNM)), two haloketones (1,1-dichloro-2-propanone (1,1-DCP) and 1,1,1-trichloro-2-propanone (1,1,1-TCP)), and three I-THMs (dichloroiodomethane (DCIM), diiodochloromethane (DICM), and triiodomethane (TIM)) were obtained from Sigma-Aldrich, Agilent, and Toronto Research Chemicals. Other chemicals used in this study are listed in Text S1.

### DBP Formation Experiments

20 mL of regular or saline wastewater (Table S1, Text S1) was buffered with phosphate to the desired pH and spiked with 100 μM of PAA, PFA, or NaOCl in amber glass vials. It is worth noting that these three disinfectants provide comparable bacterial inactivation at the same dosage as demonstrated by our recent study.^38^ The reactors were protected from ambient light, stirred vigorously, and sealed with caps with a headspace less than 5 mL. The reaction was quenched by excess sodium thiosulfate (Na_2_S_2_O_3_) after a defined reaction time.

### Batch Kinetic Experiments

The reactivities of PAA and PFA with halides, HOBr/BrO^–^, and HOI/IO^–^ was investigated. The reactions between PAA and halides have been studied previously.^43^ Thus, in this study, we investigated (i) PFA decay with and without excess Cl^–^/Br^–^, (ii) iodophenol formation in the PFA/I^–^/phenol system, (iii) BrO_3_^–^ formation in POA/HOBr systems, and (iv) IO_3_^–^ formation in POA/I^–^ systems. These experiments yielded results to simulate the rate constants of POAs with Cl^–^/Br^–^, I^–^, HOBr/BrO^–^, and HOI/IO^–^, respectively. The employed experimental conditions are summarized in Table S3.

The reaction solutions were phosphate-buffered, magnetically stirred, and measured for pH right after oxidant addition, as well as throughout the experiments. Periodically, 1 mL samples were taken. The samples in group (ii) was quenched by Na_2_S_2_O_3_, while samples for experiments (iii) and (iv) were quenched by excess phenol. It is worth noting that although phenol has been shown to be not highly effective for elimination of PAA and PFA, we found the other quenching agents, including Fe(II), cysteine, ascorbic acid, and methionine, all affected BrO_3_^–^ and/or IO_3_^–^ measurement by reducing them to Br^–^/I^–^ (data not shown). Therefore, we chose to use phenol (20 mM) to quench HOBr and HOI. Even if there were residual PAA/PFA in the samples, the measurement of BrO_3_^–^ and IO_3_^–^ could not be affected due to the rapid scavenging of their precursors (i.e., HOBr, HOI) by phenol.

### Kinetic Model Simulation

Kinetic modeling was performed to simulate the unknown rate constants of POAs with halides, HOBr/BrO^–^, and HOI/IO^–^ (Table 2, see discussion later), using the “FIT:2:3:FITDATA.TXT” command in Kintecus 4.55.31. Then, HOBr and HOI levels in POA/halide systems were predicted by the kinetic model with the reactions listed in Table 2.

**Table 2:** Principal Reactions in the POA Oxidation of Halide-Containing Ammonia-Free Water

		apparent rate constant^a^			
no.	reaction	pH 5.5	pH 7.1	pH 7.8	ref
R1	PFA → products	(3.3 ± 0.3) × 10^–2^	(4.7 ± 0.3) × 10^–2^	(7.4 ± 0.3) × 10^–2^	Figure 2, Figure S2
	(a) PFA + H_2_O → H_2_O_2_ + FA^b^	(3.3 ± 0.3) × 10^–2^	(1.8 ± 0.4) × 10^–2^	(1.6 ± 0.2) × 10^–2^	Figure 2
	(b) PFA → CO_2_ + H_2_O	(0.3 ± 0.0) × 10^–2^	(2.9 ± 0.6) × 10^–2^	(5.8 ± 0.8) × 10^–2^	Figure 2
	(c) 2 PFA → 2 FA + O_2_	negligible when [PFA]_0_ < 200 μM			Figure S2
R2	H_2_O_2_ + FA → PFA + H_2_O	negligible when [H_2_O_2_]_0_ = [HCOO^–^]_0_ < 1 mM			not shown
R3	PFA + Cl^–^ → HOCl/ClO^–^ + FA		negligible		Figure S5
R4	PFA + Br^–^ → HOBr/BrO^–^ + FA	1.12 ± 0.24	1.03 ± 0.11	0.35 ± 0.09	Figure 3
R5	PFA + I^–^ → HOI/IO^–^ + FA	(3.18 ± 0.19) × 10^3^	(2.48 ± 0.35) × 10^3^	(1.34 ± 0.09) × 10^3^	Figure 4
R6	PFA + HOBr/BrO^–^ → BrO_2_^–^ + FA		negligible		Figure 5a
R7	PFA + HOI/IO^–^ → IO_2_^–^ + FA		1.20 × 10^2^		Figure 5b
R8	PFA + IO_2_^–^ → IO_3_^–^ + FA		1.00 × 10^3^		assumed
R9	PFA + H_2_O_2_ → H_2_O + FA + O_2_	negligible	negligible	(3.16 ± 0.28) × 10^–1^	Figure 2
R10	PFA + EfOM → products		slow		not shown
R11	PAA + Cl^–^ → HOCl/ClO^–^ + AA		negligible		(43)
R12	PAA + Br^–^ → HOBr/BrO^–^ + AA^c^	2.40 × 10^–1^	2.20 × 10^–1^	1.70 × 10^–1^	(43), Text S3
R13	PAA + I^–^ → HOI/IO^–^ + AA	4.19 × 10^2^	3.89 × 10^2^	3.00 × 10^2^	(43), Text S3
R14	PAA + HOBr/BrO^–^ → BrO_2_^–^ + AA		negligible		Figure 5a
R15	PAA + HOI/IO^–^ → IO_2_^–^ + AA		1.60 × 10^1^		Figure 5b
R16	PAA + IO_2_^–^ → IO_3_^–^ + AA		1.00 × 10^3^		assumed
R17	PAA + EfOM → products		slow		not shown
R18	HOBr/BrO^–^ + H_2_O_2_ → Br^–^	6.03 × 10^2^	2.36 × 10^4^	1.09 × 10^5^	(43), Text S3
R19	HOI/IO^–^ + H_2_O_2_ → I^–^	1.59 × 10^2^	6.32 × 10^3^	3.16 × 10^4^	(52), Text S3
R20	HOBr/BrO^–^ + I^–^ → HOI/IO^–^ + Br^–^	5.00 × 10^9^	4.99 × 10^9^	4.55 × 10^9^	(47), Text S3
R21	HOI/IO^–^ + HOBr/BrO^–^ → IO_2_^–^ + Br^–^		7.40 × 10^2^		(47)
R22	HOBr/BrO^–^ + IO_2_^–^ → IO_3_^–^ + Br^–^		5.00 × 10^3^		assumed
R23	HOBr/BrO^–^ + EfOM → products		3.00 × 10^1^		(57)
R24	HOI/IO^–^ + EfOM → products		3.00 × 10^1^		assumed

a The units for apparent rate constants: R1 in min^–1^, R2–R24 in M^–1^ s^–1^.

b FA = formic acid/formate, AA = acetic acid/acetate

c The self-decay of PAA,^51^ HOBr/BrO^–^,^46^ and HOI^49^ are all negligible at the concentrations employed in this study ([PAA] < 200 μM, [HOBr/BrO^–^] < 400 μM, [HOI/IO^–^] < 0.3 μM).

### Analytical Methods

The concentrations of PAA and PFA were measured following the KI-DPD method using a UV–visible spectrophotometer detailed in our previous study.^38^ The total concentrations of PFA and H_2_O_2_ were determined by a horseradish peroxidase-2,2′-azino-bis(3-ethylbenzothiazoline-6-sulfonic) acid (HRP-ABTS) method which was validated in our previous study,^58^ and the H_2_O_2_ concentration was calculated by subtracting the PFA concentration from the total peroxides.

Iodophenols were analyzed using high-performance liquid chromatography equipped with a diode-array detector (HPLC-DAD) (Text S2). Anions (i.e., Cl^–^, Br^–^, I^–^, BrO_3_^–^, and IO_3_^–^) were measured by ion chromatography (Text S2). The DBPs were measured by a gas chromatograph equipped with an electron capture detector (GC-ECD) after liquid–liquid extraction of DBPs from water by 2 mL of methyl *tert*-butyl ether (MtBE, >99.8% purity), with 1,2-dibromopropane as the internal standard, following the protocol reported by Xu et al.^35^

## Results and Discussion

### DBP Formation during Disinfection by PAA, PFA, and Chlorine

DBP formation by the addition of PAA, PFA, and free chlorine was, for the first time, compared in the same water matrix in this study. As expected, chlorine induced considerable DBP formation in regular wastewater ([Cl^–^] = 5 mM, [Br^–^] = 9 μM, [I^–^] = nondetectable), among which THM4 and DCAN accounted for the major part (Figure S1). Although HAAs and HAMs were not measured in our study, their formation could be expected due to their correlation with THMs^59^ and HANs,^22^ respectively. The DBP formation by dosing free chlorine (NaOCl) should be ascribed to both chlorination and chloramination due to the presence of NH_4_^+^ (3.49 mg/L as N) in the wastewater (Table S1). Herein, we will use the word “chlorine” to refer to the overall effect of free and combined chlorines in the matrices. In contrast to chlorine, PAA and PFA produced little halogenated DBPs (Figure S1), suggesting that DBP formation through POA oxidation of halides was not significant at regular halide levels in typical domestic wastewater.

Subsequently, we dosed additional halides to the wastewater to create a saline matrix ([Cl^–^] = 400 mM, [Br^–^] = 500 μM, [I^–^] = 0.3 μM),^43,60,61^ which demonstrated the worst-case scenarios for Br-/I-DBP formation. There are several possible scenarios of saline wastewaters that could be subject to oxidation and/or disinfection: (1) combination of shale gas wastewater, produced by hydraulic fracturing and horizontal drilling for natural gas production, into wastewater treatment plants (WWTPs),^62^ (2) chemical oxidation treatment of ballast wastewater in ocean-going vessels,^43^ (3) application of seawater to flush toilets in fresh-water-stressed coastal cities,^60,61^ (4) decentralized treatment of urine,^63−65^ and (5) reclamation of high-salinity streams along with wastewater.^66^ The evaluation of DBP formation in the saline water matrix in this study could assist in reasonable oxidant selection for the aforementioned scenarios. While the real saline wastewaters may differ to some degree from the saline sample in this study (e.g., in organic matter compositions), our main objective to compare the DBP formation trends of POAs and chlorine should not be affected.

**Figure 1:**
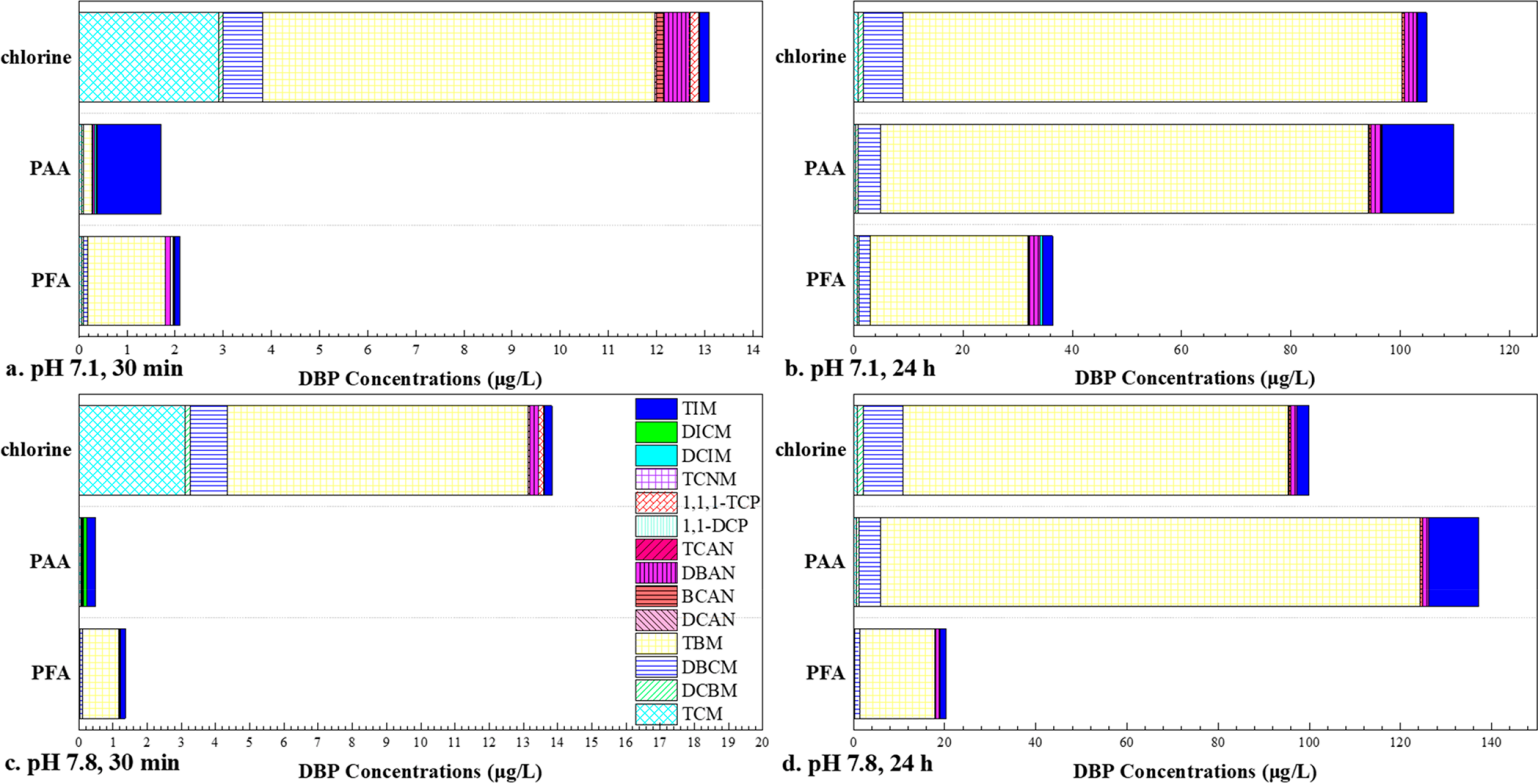
Production of 14 DBPs during disinfection of saline wastewater. Experimental conditions: [oxidant]_0_ = 100 μM, [phosphate buffer] = 10 mM, temperature 23 ± 2 °C, PAA and PFA both contained 60 μM H_2_O_2_. Saline wastewater parameters: [Cl^–^] = 400 mM, [Br^–^] = 500 μM, [I^–^] = 0.3 μM, [NH_4_^+^] = 3.49 mg/L as N, COD = 38.48 mg/L.

As shown in Figure 1a,c, during 30 min of oxidation of the synthetic saline wastewater, chlorine resulted in the highest total DBP concentration, where THM4 were the major contributors. Although PAA generated much less DBPs, it produced more TIM than chlorine at both pHs (7.1 and 7.8). The shift from THM4 to I-THMs during PAA oxidation indicated a higher HOI exposure level, which is probably associated with other I-DBP formation. Although the overall toxicity was not quantitatively calculated due to the large unknown portion of halogenated compounds, considering the high toxicity of I-DBPs,^67^ PAA probably resulted in a higher overall toxicity than chlorine in the saline wastewater, which challenges the intention to replace chlorine by PAA for DBP control in saline matrix. On the contrary, PFA controlled most DBPs effectively, only with modest TBM formation.^48,50^

As for the long-term (24 h) disinfection, relevant to ballast wastewater oxidation in ocean-going vessels, we surprisingly found that PAA led to a higher total DBP concentration than that of chlorine (Figure 1b,d). Although PAA slightly reduced the formation of chlorinated DBPs (Cl-DBPs), it enhanced the formation of most Br- and I-DBPs, including TBM, BCAN, and I-THMs. In particular, PAA triggered TIM formation at 13.15 and 10.81 μg/L at pH 7.1 and 7.8, respectively, whereas chlorine only resulted in 1.69 and 2.65 μg/L. Notably, 24 h PAA oxidation gave rise to an ∼25% iodine incorporation into I-THMs, which is higher than that reported for chloramination (<20%) at similar iodide levels (24 h, [NH_2_Cl] = 15 μM).^47^ Interestingly, contrary to PAA, PFA controlled all tested DBPs successfully during long-term disinfection.

To sum up, these results suggest that replacing chlorine by either PAA or PFA could alleviate DBP problems in regular wastewater effluents; however, with elevated halide concentrations, PFA remarkably outperformed PAA and chlorine for DBP control. Particularly, PAA induced an unwanted shift from Cl-DBPs to Br-/I-DBPs in saline wastewater, which can potentially make the disinfected effluents even more toxic than the chlorinated ones. It should be noted that, due to the presence of ammonia (3.49 mg/L as N) in the saline wastewater of this study, the generated HOCl/HOBr could be rapidly converted to corresponding halamines that could be major species contributing to Cl- and Br-DBP formation, while HOI was always the major iodinating agent due to the lack of reactivity with ammonia.^48,50^ Overall, the high ammonia concentration used in this study may inhibit the formation of carbonaceous Cl- and Br-DBPs (e.g., THM4) for POAs as well as free chlorine. The DBP formation potential of free chlorine could be particularly mitigated by ammonia due to direct and rapid consumption of the free chlorine oxidant. In fully nitrified wastewater (low ammonia levels), the advantages of POAs for DBP control (over free chlorine) may be more significant. However, we still expect PFA to outperform PAA for DBP control regardless of ammonia levels (see later discussion).

### Reactivity of POAs with Water Matrices

To understand DBP formation by PAA and PFA, we first investigated their reactivity with the major components in the saline wastewater matrix, i.e., Br^–^, I^–^, NH_4_^+^, and EfOM.

#### Reactivity of PAA with Water Matrices

The reactivity between PAA and halides has been reported by Shah et al.^43^ and hence was not repeated in this study (Table 2, R11–R13). Additionally, we found the 30 min PAA decay in the regular wastewater and in a NH_4_^+^ solution (10 mM) were both less than 5% at pH 7.1 (data not shown); thus, the PAA self-decay and PAA consumption by NH_4_^+^ and EfOM could be neglected under the experimental conditions.

#### PFA Self-Decay

**Figure 2:**
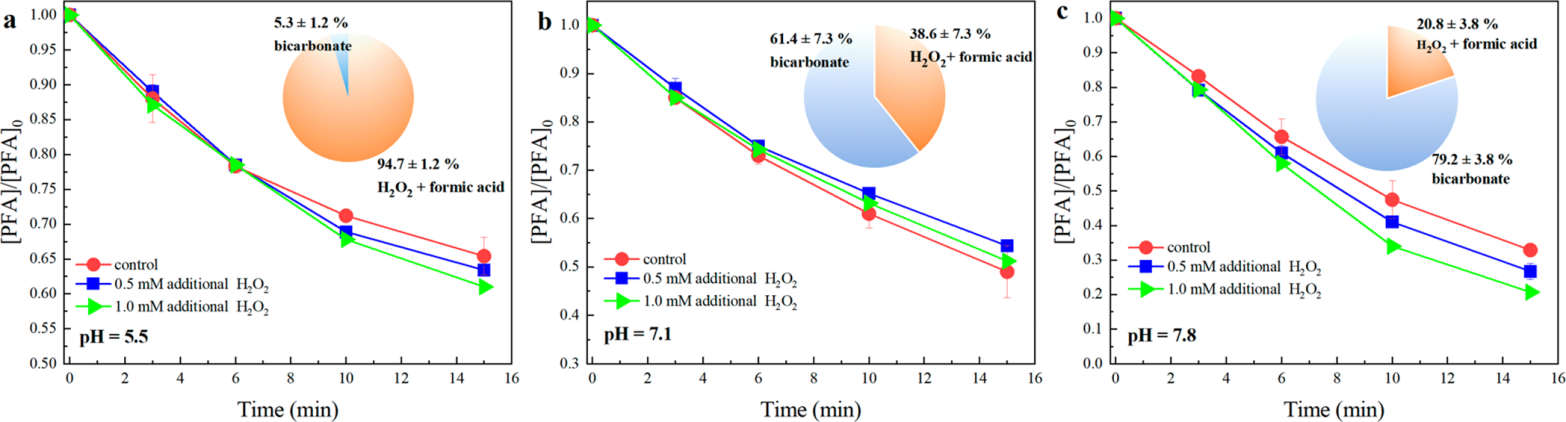
PFA decay and the percentage of conversion to H_2_O_2_ at pH 5.5 (a), 7.1 (b), and 7.8 (c). Experimental conditions: [PFA]_0_ = 100 μM, [phosphate buffer] = 10 mM, temperature 23 ± 2 °C. Error bars represent standard deviations between parallel experiments.

Unlike PAA, PFA decomposition is relatively fast and has to be studied. First, we found the change of initial PFA concentration from 50 to 200 μM did not affect PFA self-decay (Figure S2), and hence, the PFA self-decay was fitted into a pseudo-first-order kinetic model (eqs 1 and 2)
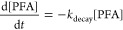
1
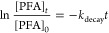
2where *k*_decay_ is the first-order rate constant for PFA self-decay (in min^–1^), [PFA] is the concentration of PFA (in M), and *t* is the reaction time (in min). *k*_decay_ increased from (3.3 ± 0.3) × 10^–2^ to (7.4 ± 0.3) × 10^–2^ min^–1^ as the pH increased from 5.5 to 7.8. As the bimolecular PFA decay has been ruled out (when [PFA] < 200 μM), PFA decay could be ascribed to R1a, R1b, and/or R9 (Table 2).^41^ First, the possible reaction between PFA and H_2_O_2_ (R9) was examined by adding additional H_2_O_2_ to the PFA solution, and additional H_2_O_2_ only affected PFA self-decay at pH 7.8 (Figure 2 and Table 2 R9). Then, relative contributions from R1a and R1b to PFA self-decay were differentiated by measuring the change in the H_2_O_2_ concentration during PFA self-decay. We found that the H_2_O_2_ pathway (Table 2, R1a) dominated PFA self-decay at pH 5.5 (94.7 ± 1.2%), while the fraction of the bicarbonate pathway (Table 2, R1b) rose remarkably with the increase in pH (i.e., from 5.3 ± 1.2% at pH 5.5 to 79.2 ± 3.8% at pH 7.8) (Figure 2).^41^ The corresponding rate constants for the two self-decay pathways (R1a,b) at three tested pHs were calculated as shown in Table 2. In contrast, the PAA self-decay was negligible within 30 min.

#### Reactivity of PFA with Water Matrices

**Figure 3:**
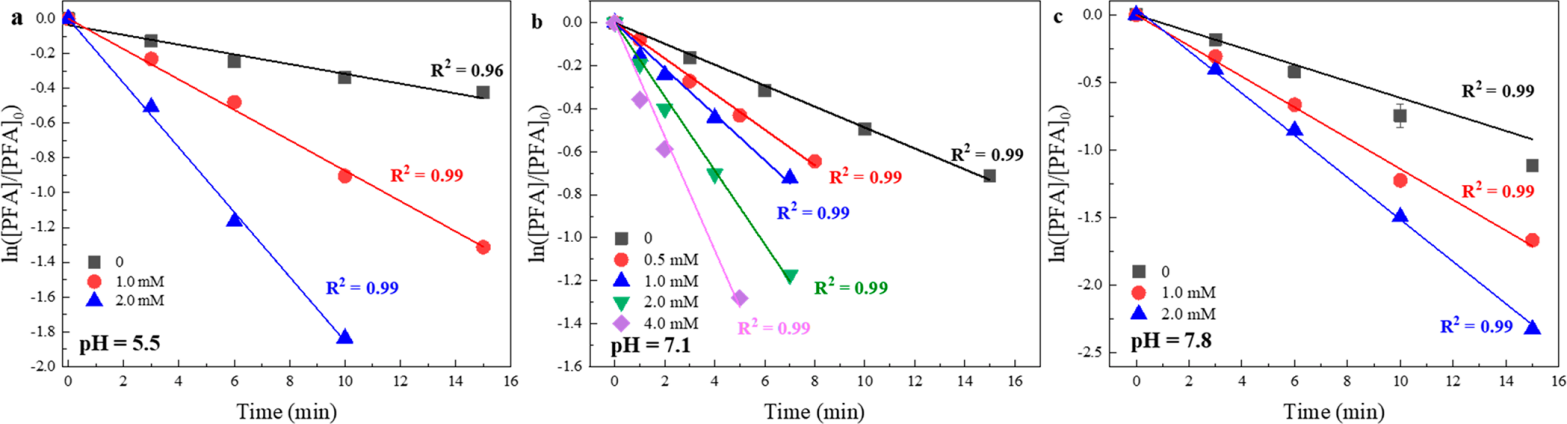
Impact of bromide (0–4.0 mM) on PFA decay. Experimental conditions: [phosphate buffer] = 10 mM, [PFA]_0_ = 100 μM, [H_2_O_2_ (additional)]_0_ = 1.0 mM, temperature 23 ± 2 °C. Error bars represent standard deviations between parallel experiments, and the solid lines represent the linear regression.

The reactivity of inorganic constituents with PFA was examined by testing their effects on the PFA decay. First of all, the presence of Br^–^ significantly accelerated PFA self-decay (Table 2, R4) (Figure 3); for those experiments an excess amount of Br^–^ was applied to achieve a pseudo-first-order reaction. We also applied additional H_2_O_2_ to reduce the produced HOBr back to Br^–^, which kept a constant Br^–^ concentration and avoided the interference of HOBr during the PFA measurement by DPD (Table 2, R18). Then, the apparent second-order rate constants were calculated by a pseudo-first-order model (eqs 3 and 4).
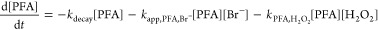
3
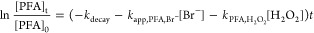
4where *k*_decay_ is the PFA self-decay rate constant (Table 2, R1) (in s^–1^), *k*_app,PFA,Br^–^_ is the apparent second-order rate constant for the reaction between PFA and Br^–^ (in M^–1^ s^–1^),[PFA] and [Br^–^] are the concentrations of PFA and Br^–^, respectively (in M), *k*_app,FA,H_2_O_2__ is the apparent second-order rate constant for the reaction between PFA and H_2_O_2_ (Table 2, R9) (in M^–1^ s^–1^) (ote that this reaction is negligible at pH 5.5 and 7.1, but non-negligible at pH 7.8), [H_2_O_2_] is the concentration of additionally spiked H_2_O_2_ (i.e., 1.0 mM), and *t* is the reaction time (in s). As a result, the apparent second-order rate constants between PFA and Br^–^ (*k*_app,PFA,Br^–^_, Table 2 R4) were determined to be 0.35–1.12 M^–1^ s^–1^ in the pH range 5.5–7.8 and are approximately linear to the fraction of protonated PFA (p*K*_a_ = 7.3^41^) at each pH (Figure S3a and Text S3), suggesting that only the protonated PFA was the reactive form for oxidation of Br^–^.

**Figure 4:**
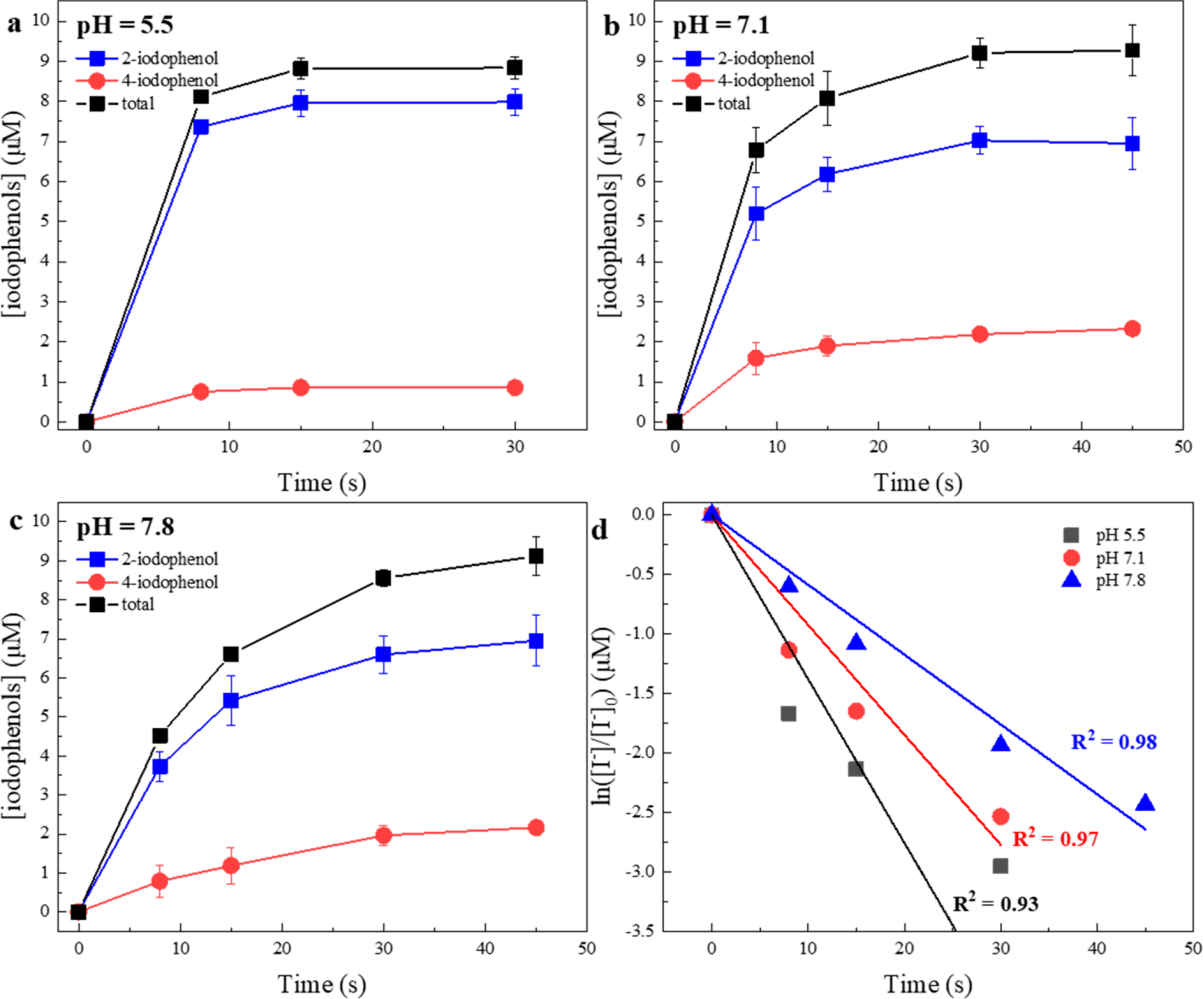
Production of iodophenols from iodide oxidation by PFA (a–c) and the corresponding iodide loss (d). Experimental conditions: [phosphate buffer] = 10 mM, [iodide]_0_ = 10 μM, [PFA]_0_ = 50 μM, [phenol]_0_ = 4.0 mM, temperature 23 ± 2 °C. Error bars represent standard deviationdbetween parallel experiments, and the solid lines in (d) represent the linear regression modeling.

The above experimental approach for Br^–^ could not be applied to study the reaction of PFA with I^–^, because excess I^–^ consumed PFA extremely rapidly. Alternatively, we used iodophenol formation to quantify HOI formation and indicate I^–^ loss,^52^ where PFA was dosed in excess to I^–^ to enable the first-order model, and phenol was dosed in excess as well to react with all the HOI/IO^–^ and minimize IO_3_^–^ formation. The iodophenol formation and I^–^ loss (Figure 4) can be described by eqs 5 and 6.
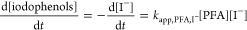
5
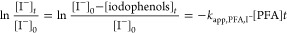
6where [iodophenols] is the total concentration of 2-, and 4-iodophenol (in M), *k*_app,PFA I^–^_ is the apparent second-order rate constant for the reaction between PFA and I^–^ (in M^–1^ s^–1^), [PFA] and [I^–^] are the concentrations of PFA and I^–^, respectively (in M), and *t* is the reaction time (in s). As a result, the rate constants between PFA and I^–^ (*k*_app,PFA,I^–^_, Table 2 R4) were determined to be (1.34–3.18) × 10^3^ M^–1^ s^–1^ in the pH range 5.5–7.8 and are linearly related to PFA protonation (Figure S3b and Text S3).

Finally, we found Cl^–^, NH_4_^+^, EfOM, and phenols all had very limited impacts on PFA self-decay (Figures S4–S7). Thus, R3 and R10 could be neglected in the kinetic model for the PFA oxidation of saline wastewater.

Interestingly, the reaction of PFA with halides was 4–5 times faster than that of PAA at each pH. As the initial coexistent H_2_O_2_ concentrations in PAA and PFA solutions were similar for the DBP formation experiments, PFA would be expected to result in higher HOBr/HOI levels and more Br- and I-DBP formation. However, PFA oxidation produced much fewer DBPs than PAA in halide-containing water, prompting us to further investigate and develop a comprehensive kinetic model to elucidate this phenomenon.

### Understanding the Distinct DBP Formation Behaviors of PAA and PFA

To understand why PFA controlled DBP formation better in halide-containing water despite its higher reactivity with halides, we proposed three hypotheses and examined them one by one.

#### Reducing Effects of Formate and Acetate

PAA and PFA solutions contain considerable amounts of formate and acetate, respectively. Particularly, formate is a weak reducing agent due to the formaldehyde moiety. Therefore, it was possible that coexistent formate reduced HOBr/HOI and controlled DBP formation. However, this possibility is ruled out by the minimal impacts of formate and acetate on HOBr/HOI decay (Figure S8).

#### Reaction between HOBr and HOI

It has been well-documented that HOBr could oxidize HOI/IO^–^ to produce BrO_3_^–^ and IO_3_^–^ (Table 2, R21 and R22).^48^ Thus, if large amounts of HOBr/BrO^–^ and HOI/IO^–^ are produced by PFA, they can be canceled out into nonreactive forms, which hence controls DBP formation. To test this hypothesis, we dosed the oxidants into synthetic wastewater containing only 0.3 μM I^–^ [without Br^–^]. Nonetheless, PAA still produced more TIM than did PFA and chlorine (Figure S9).

#### Production of BrO_3_^–^ and IO_3_^–^

**Figure 5:**
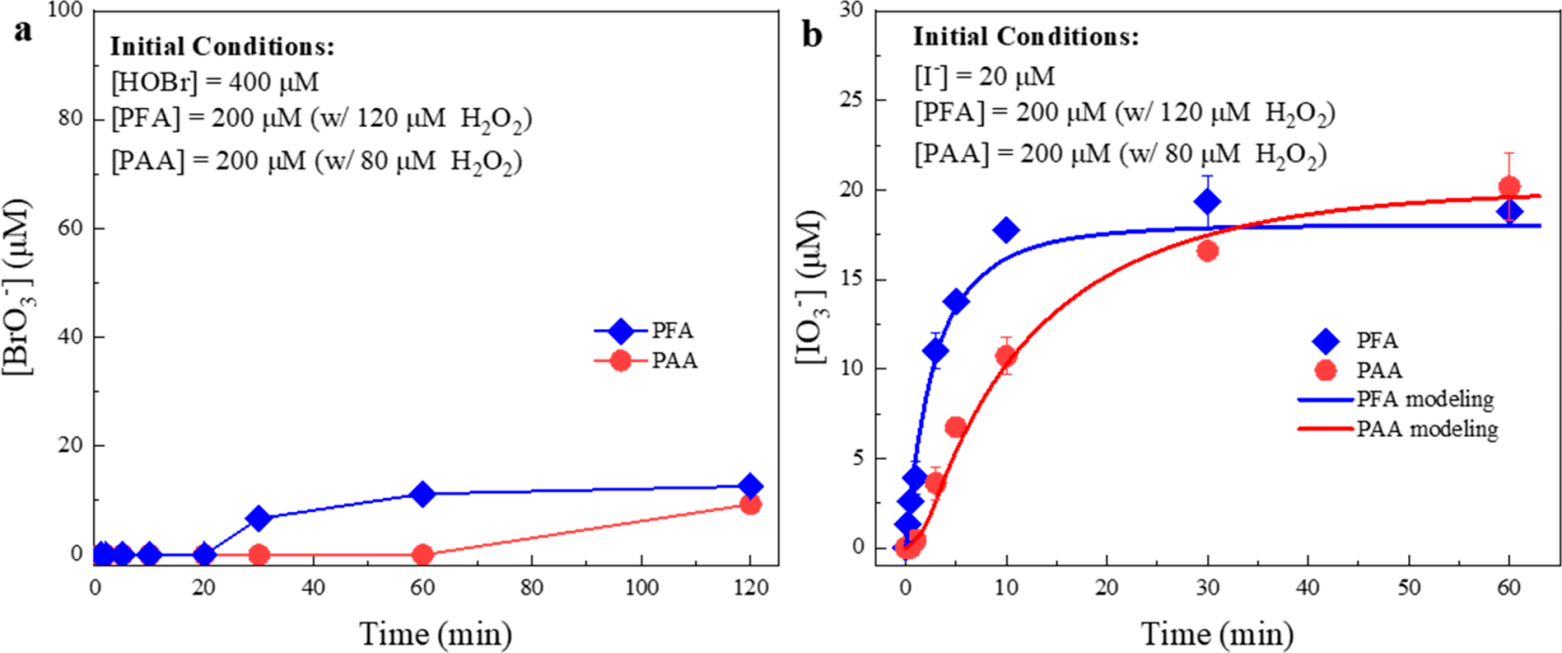
Production of bromate in POA/HOBr systems (a) and production of iodate from POA/iodide systems (b). Experimental conditions: [phosphate buffer] = 10 mM, pH = 7.1, temperature 23 ± 2 °C. Error bars represent standard deviations between parallel experiments.

Finally, as mentioned above, the byproducts will shift from organic Br- and I-DBPs to BrO_3_^–^ and IO_3_^–^, respectively, if POAs further oxidize HOBr/BrO^–^ and HOI/IO^–^. The oxidation of HOBr/BrO^–^ and HOI/IO^–^ typically proceeds through two-electron transfer and produces BrO_2_^–^ (Table 2, R6 and R14) and IO_2_^–^ (Table 2, R7 and R15), which will be rapidly oxidized to BrO_3_^–^ and IO_3_^–^, respectively (Table 2, R8 and R16, *k* assumed to be ∼10^3^ M^–1^ s^–1^).^48,50,52^ Thus, to further understand DBP formation by PAA and PFA, we simulated the rate constants for R6 and R14 in the POA/HOBr system and R7 and R15 in the POA/I^–^ system at pH 7.1. HOBr is easy to synthesize and relatively stable.^57^ Therefore, we mixed preprepared HOBr and POAs at high concentrations to maximize the BrO_3_^–^ formation. However, less than 2.5% conversion from HOBr to BrO_3_^–^ was observed during the 2 h interaction (Figure 5a). It is noteworthy that in the above experiment, we already tried intentionally to maximize BrO_3_^–^ formation through spiking of 200 μM POAs and 400 μM HOBr. In fact, the HOBr concentration did not exceed 0.2 μM during POA oxidation of saline water, which was verified by the DPD measurement. Therefore, the reactions between HOBr and POAs (Table 2, R6 and R14) should be negligible during POA oxidation of bromide-containing water.

Contrary to HOBr, HOI could not be presynthesized due to its self-decay (note that this self-decay is negligible when [HOI] < 0.3 μM^49^ and was thus not incorporated into the kinetic model). Thus, we mixed excess POAs with I^–^ to test IO_3_^–^ formation (Figure 5b). Significant IO_3_^–^ formation was observed for both POAs. By kinetic model simulation, we found that PFA and PAA oxidized HOI/IO^–^ at 1.20 × 10^2^ M^–1^ s^–1^ (R7, RMSD = 0.118) and 1.60 × 10^1^ M^–1^ s^–1^ (R15, RMSD = 0.133) at pH 7.1, respectively.

The different reactivities of PAA and PFA toward HOI/IO^–^ explain why PFA controls I-DBP formation better even if it oxidizes I^–^ faster. PFA oxidizes HOI/IO^–^ into the nonreactive IO_3_^–^ rapidly and hence controls I-DBP formation, while PAA exhibits rather slow oxidation of HOI and thus induces accumulation of HOI and production of I-THM. Even chlorine produces fewer I-DBPs than PAA, which is probably due to the faster oxidation of HOI/IO^–^ by chlorine (Table 1) and competition of susceptible precursors with HOI.

### Modeling the Oxidant Concentrations

So far, the inhibition of I-DBP formation by PFA has been attributed to its fast oxidation of HOI/IO^–^ into IO_3_^–^. However, as both PAA and PFA could hardly oxidize HOBr/BrO^–^, PFA is expected to produce more Br-DBPs than PAA due to its faster oxidation of Br^–^ to HOBr, which is inconsistent with the DBP formation results during 24 h disinfection (Figure 1b,d). Therefore, we built up a comprehensive kinetic model with all of the relevant rate constants retrieved from previous studies or simulated in this study (Table 2).

To systematically compare PAA and PFA oxidation, we simulated oxidant levels with the kinetic model in a representative halide-containing water matrix ([EfOM] = 1.0 mM as C, [Br^–^]_0_ = 0.5 mM, [I^–^]_0_ = 0.2 μM, no NH_4_^+^) with two simplifications justified as below. First, the presence of NH_4_^+^ can significantly complicate the model by incorporating the reactions of NH_2_Cl, NH_2_Br, NHBrCl, and NHCl_2_. The kinetic model can be exploited with the addition of halamine reactions,^48^ if needed; however, we believe the involvement of NH_4_^+^ will not change the major conclusion on PAA/PFA comparison. Cl^–^ is not included because it could not change the modeling results due to its negligible reactions with POAs. Moreover, we would clarify that HOBr and HOI are not the only halogenating agents in the system because their speciation results in exotic electrophile formation (i.e., BrCl, BrOCl, Br_2_O, H_2_OI^+^, and ICl).^68^ Despite their low concentrations (at least 4 orders of magnitude lower than those of HOBr or HOI at pH 7.1–7.8), these species have non-negligible contributions to DBP formation due to their high reactivity. However, as these species always reach rapid equilibrium with their precursors (HOBr or HOI),^68^ the HOBr/HOI levels would not be affected and the overall DBP formation should still be proportional to HOBr/HOI levels. Overall, we believe the kinetic simulation in this representative matrix could provide valuable insight into PAA/PFA comparison, despite these acceptable simplifications.

**Figure 6:**
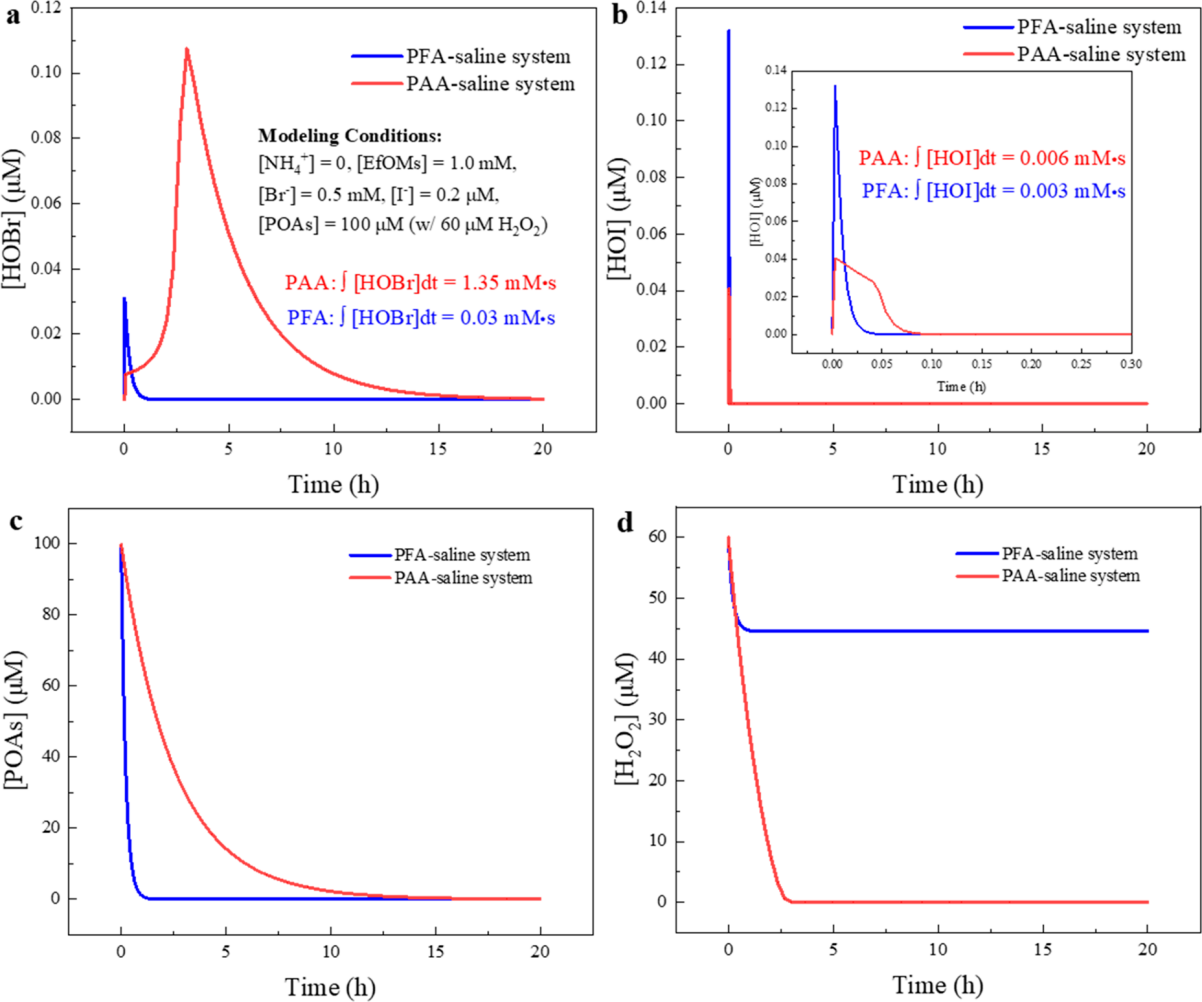
Kinetic simulation for PAA and PFA oxidation of ammonia-free saline water. The cumulative exposure to HOBr and HOI was calculated by integrating [HOBr] or [HOI] over time. Simulation time = 20 h.

Our model shows that PFA produces more HOBr and HOI than PAA in the short term (Figure 6a,b). However, the HOBr and HOI levels in the PFA/halide system reach the peak points at ∼0.88 and 0.18 h, respectively; then their concentrations decrease and are always lower than those with PAA in the long term. These simulation results concur with the DBP formation experiments, where the overall higher HOBr/HOI concentrations during PAA oxidation gave rise to its higher Br-/I-DBP formation in 24 h, whereas the rapid HOBr generation by PFA yielded more Br-DBPs in the first 30 min (Figure 1).

Overall, the gradually decreasing HOBr/HOI levels during PFA oxidation remarkably control the DBP formation and could be attributed to three mechanisms. First, as indicated by the model, PFA is quickly consumed by the self-decay and faster reactions with halides, while the PAA decay is much slower (Figure 6c). The elimination of PFA cuts off the source for HOBr and HOI, while the relatively stable PAA maintains the oxidation of halides and the formation of HOBr/HOI. Second, H_2_O_2_ is the major reductant for HOBr/HOI in the POA disinfection processes and benefits the control of Br- and I-DBPs. Although we intentionally controlled the initial H_2_O_2_ in PAA and PFA at the same concentration (for both DBP experiments and kinetic simulation), the model showed that the H_2_O_2_ in the PFA system could not be totally consumed (Figure 6d), while the H_2_O_2_ in PAA is depleted within 3 h. Briefly, in the PFA system, the depletion of the HOBr/HOI source (i.e., PFA) inhibits H_2_O_2_ consumption by suppressing R18 and R19 (Table 2), and additional H_2_O_2_ could be released during PFA self-decay (Table 2, R1a). In contrast, the relatively stable PAA maintains halide oxidation and the catalytic depletion of H_2_O_2_ (Table 2, R18 and R19). The higher H_2_O_2_ level during PFA oxidation definitely contributes to the HOBr/HOI reduction and DBP control. Third, for I-DBPs, PFA oxidizes HOI/IO^–^ into IO_3_^–^ faster than PAA and hence limits the iodination of EfOM.

To sum up, the kinetic model shows that PFA’s faster decay, slow release of H_2_O_2_, and faster oxidation of HOI/IO^–^ all limit the HOBr/HOI levels and control Br- and I-DBPs formation. In contrast, the stability of PAA results in continuous halide oxidation and catalytic depletion of H_2_O_2_, leading to higher HOBr/HOI levels and intensive DBP formation.

### Environmental Significance

This study, for the first time, systematically compares the DBP formation potential of PAA, PFA, and chlorine. Both PAA and PFA showed a satisfactory DBP control capacity in regular wastewater. However, only PFA controlled DBP formation in saline wastewater, while PAA produced more Br- and I-DBPs than chlorine after 24 h oxidation.

Additionally, we developed a kinetic model for POA/halide systems. The reactivity of PAA and PFA toward halides can be compared with those of other oxidants (Table 1). PAA joins monochloramine as a significant contributor to I-DBPs, while PFA exhibits several properties beneficial for DBP control, including (i) low reactivity with HOBr/BrO^–^, (ii) self-decay and slow release of H_2_O_2_, and (iii) efficient oxidation of HOI to IO_3_^–^. As BrO_3_^–^ formation has been reported as a problem for both ferrate(VI)^53^ and ozone,^32^ PFA seems to be the only disinfectant that produces neither BrO_3_^–^ or I-DBPs in saline effluents. So far, in-depth studies have shown PFA to be more effective than PAA for pathogen inactivation^38^ and DBP control (this study). Hence, PFA is a strong candidate for disinfection of halide-containing water; however, on-site generation and immediate application of PFA are required due to its fast self-decay.

## Supporting Information Available

Chemicals, analytical methods, calculation of rate constants, water quality parameters, and PFA decay experiements (PDF)

## References

[ref1] Li X. F.; Mitch W. A. Drinking Water Disinfection Byproducts (DBPs) and Human Health Effects: Multidisciplinary Challenges and Opportunities. Environ. Sci. Technol. 2018, 52 (4), 1681–1689. 10.1021/acs.est.7b05440. 29283253

[ref2] Krasner S. W.; Mitch W. A.; Westerhoff P.; Dotson A. Formation and control of emerging C- and N-DBPs in drinking water. Journal - American Water Works Association 2012, 104 (11), E582–E595. 10.5942/jawwa.2012.104.0148.

[ref3] Simpson A. M. A.; Mitch W. A. Chlorine and ozone disinfection and disinfection byproducts in postharvest food processing facilities: A review. Critical Reviews in Environmental Science and Technology 2022, 52 (11), 1825–1867. 10.1080/10643389.2020.1862562.

[ref4] Allen J. M.; Plewa M. J.; Wagner E. D.; Wei X.; Bokenkamp K.; Hur K.; Jia A.; Liberatore H. K.; Lee C. T.; Shirkhani R.; Krasner S. W.; Richardson S. D. Drivers of Disinfection Byproduct Cytotoxicity in U.S. Drinking Water: Should Other DBPs Be Considered for Regulation?. Environ. Sci. Technol. 2022, 56 (1), 392–402. 10.1021/acs.est.1c07998. 34910457

[ref5] Krasner S. W.; Mitch W. A.; McCurry D. L.; Hanigan D.; Westerhoff P. Formation, precursors, control, and occurrence of nitrosamines in drinking water: a review. Water Res. 2013, 47 (13), 4433–50. 10.1016/j.watres.2013.04.050. 23764594

[ref6] Dong H.; Cuthbertson A. A.; Richardson S. D. Effect-Directed Analysis (EDA): A Promising Tool for Nontarget Identification of Unknown Disinfection Byproducts in Drinking Water. Environ. Sci. Technol. 2020, 54 (3), 1290–1292. 10.1021/acs.est.0c00014. 31951395

[ref7] Richardson S. D. Tackling unknown disinfection by-products: Lessons learned. Journal of Hazardous Materials Letters 2021, 2, 100041 10.1016/j.hazl.2021.100041.

[ref8] McCurry D. L.; Bear S. E.; Bae J.; Sedlak D. L.; McCarty P. L.; Mitch W. A. Superior Removal of Disinfection Byproduct Precursors and Pharmaceuticals from Wastewater in a Staged Anaerobic Fluidized Membrane Bioreactor Compared to Activated Sludge. Environmental Science & Technology Letters 2014, 1 (11), 459–464. 10.1021/ez500279a.

[ref9] Dominguez Henao L.; Turolla A.; Antonelli M. Disinfection by-products formation and ecotoxicological effects of effluents treated with peracetic acid: A review. Chemosphere 2018, 213, 25–40. 10.1016/j.chemosphere.2018.09.005. 30212717

[ref10] Daiber E. J.; DeMarini D. M.; Ravuri S. A.; Liberatore H. K.; Cuthbertson A. A.; Thompson-Klemish A.; Byer J. D.; Schmid J. E.; Afifi M. Z.; Blatchley E. R. 3rd; Richardson S. D. Progressive Increase in Disinfection Byproducts and Mutagenicity from Source to Tap to Swimming Pool and Spa Water: Impact of Human Inputs. Environ. Sci. Technol. 2016, 50 (13), 6652–62. 10.1021/acs.est.6b00808. 27124361

[ref11] Lee W.-N.; Huang C.-H. Formation of disinfection byproducts in wash water and lettuce by washing with sodium hypochlorite and peracetic acid sanitizers. Food Chemistry: X 2019, 1, 100003 10.1016/j.fochx.2018.100003.

[ref12] Pruss-Ustun A.; Wolf J.; Bartram J.; Clasen T.; Cumming O.; Freeman M. C.; Gordon B.; Hunter P. R.; Medlicott K.; Johnston R. Burden of disease from inadequate water, sanitation and hygiene for selected adverse health outcomes: An updated analysis with a focus on low- and middle-income countries. Int. J. Hyg Environ. Health 2019, 222 (5), 765–777. 10.1016/j.ijheh.2019.05.004. 31088724 PMC6593152

[ref13] Mitch W. A. Tap water and bladder cancer in China. Nature Sustainability 2022, 5, 643–644. 10.1038/s41893-022-00900-0.

[ref14] Marron E. L.; Van Buren J.; Cuthbertson A. A.; Darby E.; von Gunten U.; Sedlak D. L. Reactions of alpha, beta-Unsaturated Carbonyls with Free Chlorine, Free Bromine, and Combined Chlorine. Environ. Sci. Technol. 2021, 55 (5), 3305–3312. 10.1021/acs.est.0c07660. 33565865 PMC9255599

[ref15] Marron E. L.; Mitch W. A.; Gunten U. V.; Sedlak D. L. A Tale of Two Treatments: The Multiple Barrier Approach to Removing Chemical Contaminants During Potable Water Reuse. Acc. Chem. Res. 2019, 52 (3), 615–622. 10.1021/acs.accounts.8b00612. 30821146 PMC7653687

[ref16] Marron E. L.; Prasse C.; Buren J. V.; Sedlak D. L. Formation and Fate of Carbonyls in Potable Water Reuse Systems. Environ. Sci. Technol. 2020, 54 (17), 10895–10903. 10.1021/acs.est.0c02793. 32833432 PMC7755163

[ref17] Jiang J.; Han J.; Zhang X. Nonhalogenated Aromatic DBPs in Drinking Water Chlorination: A Gap between NOM and Halogenated Aromatic DBPs. Environ. Sci. Technol. 2020, 54 (3), 1646–1656. 10.1021/acs.est.9b06403. 31909989

[ref18] Han J.; Zhang X.; Jiang J.; Li W. How Much of the Total Organic Halogen and Developmental Toxicity of Chlorinated Drinking Water Might Be Attributed to Aromatic Halogenated DBPs?. Environ. Sci. Technol. 2021, 55 (9), 5906–5916. 10.1021/acs.est.0c08565. 33830743

[ref19] Wu Y.; Wei W.; Luo J.; Pan Y.; Yang M.; Hua M.; Chu W.; Shuang C.; Li A. Comparative Toxicity Analyses from Different Endpoints: Are New Cyclic Disinfection Byproducts (DBPs) More Toxic than Common Aliphatic DBPs?. Environ. Sci. Technol. 2022, 56 (1), 194–207. 10.1021/acs.est.1c03292. 34935353

[ref20] Deborde M.; von Gunten U. Reactions of chlorine with inorganic and organic compounds during water treatment-Kinetics and mechanisms: a critical review. Water Res. 2008, 42 (1–2), 13–51. 10.1016/j.watres.2007.07.025. 17915284

[ref21] Gao J.; Proulx F.; Rodriguez M. J. Halogenated acetaldehydes in water: A review of their occurrence, formation, precursors and control strategies. Critical Reviews in Environmental Science and Technology 2019, 49 (15), 1331–1385. 10.1080/10643389.2019.1571353.

[ref22] Shah A. D.; Mitch W. A. Halonitroalkanes, halonitriles, haloamides, and N-nitrosamines: a critical review of nitrogenous disinfection byproduct formation pathways. Environ. Sci. Technol. 2012, 46 (1), 119–31. 10.1021/es203312s. 22112205

[ref23] Huang H.; Wu Q. Y.; Hu H. Y.; Mitch W. A. Dichloroacetonitrile and dichloroacetamide can form independently during chlorination and chloramination of drinking waters, model organic matters, and wastewater effluents. Environ. Sci. Technol. 2012, 46 (19), 10624–31. 10.1021/es3025808. 22950789

[ref24] Zhang R.; Meng T.; Huang C. H.; Ben W.; Yao H.; Liu R.; Sun P. PPCP Degradation by Chlorine-UV Processes in Ammoniacal Water: New Reaction Insights, Kinetic Modeling, and DBP Formation. Environ. Sci. Technol. 2018, 52 (14), 7833–7841. 10.1021/acs.est.8b00094. 29906121

[ref25] Szczuka A.; Huang N.; MacDonald J. A.; Nayak A.; Zhang Z.; Mitch W. A. N-Nitrosodimethylamine Formation during UV/Hydrogen Peroxide and UV/Chlorine Advanced Oxidation Process Treatment Following Reverse Osmosis for Potable Reuse. Environ. Sci. Technol. 2020, 54 (23), 15465–15475. 10.1021/acs.est.0c05704. 33185421

[ref26] Park S.-H.; Wei S.; Mizaikoff B.; Taylor A. E.; Favero C.; Huang C.-H. Degradation of Amine-Based Water Treatment Polymers during Chloramination as N-Nitrosodimethylamine (NDMA) Precursors. Environ. Sci. Technol. 2009, 43 (5), 1360–1366. 10.1021/es802732z. 19350904

[ref27] Spahr S.; Cirpka O. A.; von Gunten U.; Hofstetter T. B. Formation of N-Nitrosodimethylamine during Chloramination of Secondary and Tertiary Amines: Role of Molecular Oxygen and Radical Intermediates. Environ. Sci. Technol. 2017, 51 (1), 280–290. 10.1021/acs.est.6b04780. 27958701

[ref28] Vu T. N.; Kimura S. Y.; Plewa M. J.; Richardson S. D.; Marinas B. J. Predominant N-Haloacetamide and Haloacetonitrile Formation in Drinking Water via the Aldehyde Reaction Pathway. Environ. Sci. Technol. 2019, 53 (2), 850–859. 10.1021/acs.est.8b02862. 30522267

[ref29] Shi J. L.; Plata S. L.; Kleimans M.; Childress A. E.; McCurry D. L. Formation and Fate of Nitromethane in Ozone-Based Water Reuse Processes. Environ. Sci. Technol. 2021, 55 (9), 6281–6289. 10.1021/acs.est.0c07895. 33881830

[ref30] Lim S.; Shi J. L.; von Gunten U.; McCurry D. L. Ozonation of organic compounds in water and wastewater: A critical review. Water Res. 2022, 213, 118053 10.1016/j.watres.2022.118053. 35196612

[ref31] McCurry D. L.; Quay A. N.; Mitch W. A. Ozone Promotes Chloropicrin Formation by Oxidizing Amines to Nitro Compounds. Environ. Sci. Technol. 2016, 50 (3), 1209–17. 10.1021/acs.est.5b04282. 26752338

[ref32] Yang J.; Dong Z.; Jiang C.; Wang C.; Liu H. An overview of bromate formation in chemical oxidation processes: Occurrence, mechanism, influencing factors, risk assessment, and control strategies. Chemosphere 2019, 237, 124521 10.1016/j.chemosphere.2019.124521. 31408797

[ref33] Shah A. D.; Dotson A. D.; Linden K. G.; Mitch W. A. Impact of UV disinfection combined with chlorination/chloramination on the formation of halonitromethanes and haloacetonitriles in drinking water. Environ. Sci. Technol. 2011, 45 (8), 3657–64. 10.1021/es104240v. 21417331

[ref34] Chen C.; Du Y.; Zhou Y.; Wu Q.; Zheng S.; Fang J. Formation of nitro(so) and chlorinated products and toxicity alteration during the UV/monochloramine treatment of phenol. Water Res. 2021, 194, 116914 10.1016/j.watres.2021.116914. 33636667

[ref35] Xu J.; Kralles Z. T.; Dai N. Effects of Sunlight on the Trichloronitromethane Formation Potential of Wastewater Effluents: Dependence on Nitrite Concentration. Environ. Sci. Technol. 2019, 53 (8), 4285–4294. 10.1021/acs.est.9b00447. 30913390

[ref36] Xue R.; Shi H.; Ma Y.; Yang J.; Hua B.; Inniss E. C.; Adams C. D.; Eichholz T. Evaluation of thirteen haloacetic acids and ten trihalomethanes formation by peracetic acid and chlorine drinking water disinfection. Chemosphere 2017, 189, 349–356. 10.1016/j.chemosphere.2017.09.059. 28942261

[ref37] Maffettone R.; Manoli K.; Santoro D.; Passalacqua K. D.; Wobus C. E.; Sarathy S. Performic Acid Disinfection of Municipal Secondary Effluent Wastewater: Inactivation of Murine Norovirus, Fecal Coliforms, and Enterococci. Environ. Sci. Technol. 2020, 54 (19), 12761–12770. 10.1021/acs.est.0c05144. 32835477

[ref38] Wang J.; Chen W.; Wang T.; Reid E.; Krall C.; Kim J.; Zhang T.; Xie X.; Huang C. H. Bacteria and Virus Inactivation: Relative Efficacy and Mechanisms of Peroxyacids and Chlor(am) ine. Environ. Sci. Technol. 2023, 10.1021/acs.est.2c09824. PMC10690719 36995048

[ref39] Ragazzo P.; Chiucchini N.; Piccolo V.; Spadolini M.; Carrer S.; Zanon F.; Gehr R. Wastewater disinfection: long-term laboratory and full-scale studies on performic acid in comparison with peracetic acid and chlorine. Water Res. 2020, 184, 116169 10.1016/j.watres.2020.116169. 32707309

[ref40] Kim J.; Huang C.-H. Reactivity of Peracetic Acid with Organic Compounds: A Critical Review. ACS ES&T Water 2021, 1 (1), 15–33. 10.1021/acsestwater.0c00029.

[ref41] Santacesaria E.; Russo V.; Tesser R.; Turco R.; Di Serio M. Kinetics of Performic Acid Synthesis and Decomposition. Ind. Eng. Chem. Res. 2017, 56 (45), 12940–12952. 10.1021/acs.iecr.7b00593.

[ref42] Luukkonen T.; Heyninck T.; Ramo J.; Lassi U. Comparison of organic peracids in wastewater treatment: Disinfection, oxidation and corrosion. Water Res. 2015, 85, 275–85. 10.1016/j.watres.2015.08.037. 26342181

[ref43] Shah A. D.; Liu Z. Q.; Salhi E.; Hofer T.; von Gunten U. Peracetic acid oxidation of saline waters in the absence and presence of H (2) O (2): secondary oxidant and disinfection byproduct formation. Environ. Sci. Technol. 2015, 49 (3), 1698–705. 10.1021/es503920n. 25611970

[ref44] West D. M.; Wu Q.; Donovan A.; Shi H.; Ma Y.; Jiang H.; Wang J. N-nitrosamine formation by monochloramine, free chlorine, and peracetic acid disinfection with presence of amine precursors in drinking water system. Chemosphere 2016, 153, 521–7. 10.1016/j.chemosphere.2016.03.035. 27037659

[ref45] Shah A. D.; Liu Z.-Q.; Salhi E.; Höfer T.; Werschkun B.; von Gunten U. Formation of disinfection by-products during ballast water treatment with ozone, chlorine, and peracetic acid: influence of water quality parameters. Environmental Science: Water Research & Technology 2015, 1 (4), 465–480. 10.1039/C5EW00061K.

[ref46] Heeb M. B.; Criquet J.; Zimmermann-Steffens S. G.; von Gunten U. Oxidative treatment of bromide-containing waters: formation of bromine and its reactions with inorganic and organic compounds-a critical review. Water Res. 2014, 48, 15–42. 10.1016/j.watres.2013.08.030. 24184020

[ref47] Criquet J.; Allard S.; Salhi E.; Joll C. A.; Heitz A.; von Gunten U. Iodate and iodo-trihalomethane formation during chlorination of iodide-containing waters: role of bromide. Environ. Sci. Technol. 2012, 46 (13), 7350–7. 10.1021/es301301g. 22667818

[ref48] Zhu X.; Zhang X. Modeling the formation of TOCl, TOBr and TOI during chlor(am) ination of drinking water. Water Res. 2016, 96, 166–76. 10.1016/j.watres.2016.03.051. 27038586

[ref49] Bichsel Y.; von Gunten U. Oxidation of Iodide and Hypoiodous Acid in the Disinfection of Natural Waters. Environ. Sci. Technol. 1999, 33 (22), 4040–4045. 10.1021/es990336c.

[ref50] Zhai H.; Zhang X.; Zhu X.; Liu J.; Ji M. Formation of brominated disinfection byproducts during Chloramination of drinking water: new polar species and overall kinetics. Environ. Sci. Technol. 2014, 48 (5), 2579–88. 10.1021/es4034765. 24512354

[ref51] Cai M.; Sun P.; Zhang L.; Huang C. H. UV/Peracetic Acid for Degradation of Pharmaceuticals and Reactive Species Evaluation. Environ. Sci. Technol. 2017, 51 (24), 14217–14224. 10.1021/acs.est.7b04694. 29148739

[ref52] Shin J.; von Gunten U.; Reckhow D. A.; Allard S.; Lee Y. Reactions of Ferrate(VI) with Iodide and Hypoiodous Acid: Kinetics, Pathways, and Implications for the Fate of Iodine during Water Treatment. Environ. Sci. Technol. 2018, 52 (13), 7458–7467. 10.1021/acs.est.8b01565. 29856214

[ref53] Huang X.; Deng Y.; Liu S.; Song Y.; Li N.; Zhou J. Formation of bromate during ferrate(VI) oxidation of bromide in water. Chemosphere 2016, 155, 528–533. 10.1016/j.chemosphere.2016.04.093. 27153235

[ref54] Zhao X.; Salhi E.; Liu H.; Ma J.; von Gunten U. Kinetic and Mechanistic Aspects of the Reactions of Iodide and Hypoiodous Acid with Permanganate: Oxidation and Disproportionation. Environ. Sci. Technol. 2016, 50 (8), 4358–65. 10.1021/acs.est.6b00320. 27003721

[ref55] Kim J.; Du P.; Liu W.; Luo C.; Zhao H.; Huang C. H. Cobalt/Peracetic Acid: Advanced Oxidation of Aromatic Organic Compounds by Acetylperoxyl Radicals. Environ. Sci. Technol. 2020, 54 (8), 5268–5278. 10.1021/acs.est.0c00356. 32186188

[ref56] Kim J.; Zhang T.; Liu W.; Du P.; Dobson J. T.; Huang C. H. Advanced Oxidation Process with Peracetic Acid and Fe(II) for Contaminant Degradation. Environ. Sci. Technol. 2019, 53 (22), 13312–13322. 10.1021/acs.est.9b02991. 31638386

[ref57] Guo K.; Zheng S.; Zhang X.; Zhao L.; Ji S.; Chen C.; Wu Z.; Wang D.; Fang J. Roles of Bromine Radicals and Hydroxyl Radicals in the Degradation of Micropollutants by the UV/Bromine Process. Environ. Sci. Technol. 2020, 54 (10), 6415–6426. 10.1021/acs.est.0c00723. 32320225

[ref58] Wang J.; Kim J.; Ashley D. C.; Sharma V. K.; Huang C. H. Peracetic Acid Enhances Micropollutant Degradation by Ferrate(VI) through Promotion of Electron Transfer Efficiency. Environ. Sci. Technol. 2022, 56 (16), 11683–11693. 10.1021/acs.est.2c02381. 35880779

[ref59] Hua G.; Reckhow D. A.; Abusallout I. Correlation between SUVA and DBP formation during chlorination and chloramination of NOM fractions from different sources. Chemosphere 2015, 130, 82–9. 10.1016/j.chemosphere.2015.03.039. 25862949

[ref60] Yang M.; Liu J.; Zhang X.; Richardson S. D. Comparative Toxicity of Chlorinated Saline and Freshwater Wastewater Effluents to Marine Organisms. Environ. Sci. Technol. 2015, 49 (24), 14475–83. 10.1021/acs.est.5b03796. 26505276

[ref61] Yang M.; Zhang X. Halopyrroles: a new group of highly toxic disinfection byproducts formed in chlorinated saline wastewater. Environ. Sci. Technol. 2014, 48 (20), 11846–52. 10.1021/es503312k. 25236171

[ref62] Parker K. M.; Zeng T.; Harkness J.; Vengosh A.; Mitch W. A. Enhanced formation of disinfection byproducts in shale gas wastewater-impacted drinking water supplies. Environ. Sci. Technol. 2014, 48 (19), 11161–9. 10.1021/es5028184. 25203743

[ref63] Zhang R.; Sun P.; Boyer T. H.; Zhao L.; Huang C. H. Degradation of pharmaceuticals and metabolite in synthetic human urine by UV, UV/H2O2, and UV/PDS. Environ. Sci. Technol. 2015, 49 (5), 3056–66. 10.1021/es504799n. 25625668

[ref64] Luo C.; Feng M.; Sharma V. K.; Huang C. H. Oxidation of Pharmaceuticals by Ferrate(VI) in Hydrolyzed Urine: Effects of Major Inorganic Constituents. Environ. Sci. Technol. 2019, 53 (9), 5272–5281. 10.1021/acs.est.9b00006. 30933490

[ref65] Zhang R.; Yang Y.; Huang C.-H.; Li N.; Liu H.; Zhao L.; Sun P. UV/H2O2 and UV/PDS Treatment of Trimethoprim and Sulfamethoxazole in Synthetic Human Urine: Transformation Products and Toxicity. Environ. Sci. Technol. 2016, 50 (5), 2573–2583. 10.1021/acs.est.5b05604. 26840504

[ref66] Wei X.; Sanders K. T.; Childress A. E. Reclaiming wastewater with increasing salinity for potable water reuse: Water recovery and energy consumption during reverse osmosis desalination. Desalination 2021, 520, 115316 10.1016/j.desal.2021.115316.

[ref67] Dong H.; Qiang Z.; Richardson S. D. Formation of Iodinated Disinfection Byproducts (I-DBPs) in Drinking Water: Emerging Concerns and Current Issues. Acc. Chem. Res. 2019, 52 (4), 896–905. 10.1021/acs.accounts.8b00641. 30919613

[ref68] Rose M. R.; Lau S. S.; Prasse C.; Sivey J. D. Exotic Electrophiles in Chlorinated and Chloraminated Water: When Conventional Kinetic Models and Reaction Pathways Fall Short. Environ. Sci. Technol. Lett. 2020, 7 (6), 360–370. 10.1021/acs.estlett.0c00259.

