# Supplementary material for: Mechanistic Insight for Disinfection Byproduct Formation Potential of Peracetic Acid and Performic Acid in Halide-Containing Water
Source: Environ Sci Technol. 2023 Jul 25;57(47):18898–908. doi: 10.1021/acs.est.3c00670 (PMC10690735; doi:10.1021/acs.est.3c00670)
Supplement: Supplementary file 1 — es3c00670_si_001.pdf [file es3c00670_si_001.pdf]

Supporting Information

Mechanistic Insight for Disinfection Byproduct Formation Potential of Peracetic Acid and Performic Acid in Halide-Containing Water

Junyue Wang,[†] Jiale Xu,[†,#] Juhee Kim,[†] Ching-Hua Huang*,[†]

[†]School of Civil and Environmental Engineering, Georgia Institute of Technology, Atlanta, Georgia 30332, United States

[#] Current address: Department of Civil, Construction and Environmental Engineering, North Dakoda State University, Fargo, North Dakoda 58105, United States

*Corresponding Author.

E-mails: ching-hua.huang@ce.gatech.edu (Ching-Hua Huang)

Number of Pages: 16

Number of Texts: 3

Number of Tables: 3

Number of Figures: 9

Number of References: 8

Text S1. Chemicals and Reagents

Sodium hydroxide (NaOH), sulfuric acid (H_2SO_4), sodium thiosulfate ($Na_2S_2O_3$), sodium hydrogen phosphate (Na_2HPO_4), *N,N*-diethyl-*p*-phenylenediamine (DPD), sodium hypochlorite (NaOCl), phenol, 2-iodophenol, 4-iodophenol, sodium chloride (NaCl), sodium bromide (NaBr), potassium iodide (KI), potassium bromate ($KBrO_3$), potassium iodate (KIO_3), ammonium nitrate (NH_4NO_3), formic acid, acetic acid, cysteine, methionine, ascorbic acid, sodium sulfate, 1,2-dibromopropane, methy *tert*-butyl ether (MtBE) were purchased from Sigma-Aldrich or Fisher Scientific (Fair Lawn, NJ).

Deionized water (DI water) (>18 mΩ-cm) was produced from a Milli-Q water purification system (Billerica, MA). Secondary wastewater effluent, after activated sludge biological treatment and clarification, was collected from a municipal wastewater treatment plant near Atlanta, Georgia, USA. The COD and NH_4^+ concentrations of the wastewater were reported by the treatment plant. Additional halides were dosed into the regular wastewater to prepare the synthetic saline wastewater (Table S1).

Table S1. Water quality parameters for the regular and saline wastewater in this study

water quality parameters	saline wastewater	regular wastewater
Cl^- (M)	0.4	0.005
Br^- (mM)	0.5	0.009
I^- (μM)	0.3	n.d.
NH_4^+-N (mg/L as N)	3.49	3.49
COD (mg/L)	38.48	38.48
phosphorous buffer (mM)	10	10
pH	7.1 or 7.8	7.1

n.d. = non-detectable

PFA was synthesized by mixing 1.5 mL of H_2O_2 (30% w/w), 2.5 mL of formic acid (96% purity), and 0.2 mL of H_2SO_4 at 40 °C for 8 min. The density, purity, and molecular weight of formic acid was reported by the manufacture. The concentration of the H_2O_2 stock

was measured by titration methods as described in our previous study.[1] The generated PFA was stored at -4 °C and used within 2 h. The conversion rates of formic acid and H_2O_2 to yield PFA were calculated as following:

$$[\text{formic acid}]_0 = 0.96 \times \frac{1.22 \text{ g/ml}}{46 \text{ g/mol}} \times \frac{2.5 \text{ mL}}{4.2 \text{ mL}} = 15.15 \text{ M} \quad \text{(S1)}$$

$$[H_2O_2]_{\text{stock}} = 8.85 \text{ M (measured)}, [H_2O_2]_0 = 8.85 \text{ M} \times \frac{1.5 \text{ mL}}{4.2 \text{ mL}} = 3.16 \text{ M} \quad \text{(S2)}$$

$$[\text{PFA}]_{\text{final}} = 1.976 \text{ M (measured)}, [H_2O_2]_{\text{final}} = 1.126 \text{ M (measured)} \quad \text{(S3)}$$

$$\text{conversion (formic acid)} = \frac{1.976 \text{ M}}{15.15 \text{ M}} = 13.04\% \quad \text{(S4)}$$

$$\text{conversion } (H_2O_2) = \frac{1.976 \text{ M}}{3.16 \text{ M}} = 62.53\% \quad \text{(S5)}$$

Text S2. Analytical Methods

Iodophenols were analyzed using an Agilent 1100 high performance liquid chromatography equipped with an Agilent Zorbax SB–C18 column (2.1 × 150 mm, 5 μm) and a diode-array detector (HPLC-DAD). The mobile phase consisted of 40% acetonitrile and 60% DI water with 0.1% formic acid (v/v) at the flow rate of 0.3 mL/min.

Chloride, bromide, and iodide of the original wastewater were measured by ion chromatography (IC) followed by a conductivity detector. Iodate and bromate were measured by ion chromatography followed by post-column UV detection. Both IC methods employed a Dionex IonPac™ AS14A column (4 × 250 mm) and 8.0 mM Na_2CO_3/1.0 mM $NaHCO_3$ as the eluent at the flow rate of 1.0 mL/min.[2]

The DBPs were measured by gas chromatography equipped with an electron capture detector (GC-ECD) after liquid-liquid extraction by 2 mL of methyl *tert*-butyl ether (MtBE, >99.8% purity) with 1,2-dibromopropano as the internal standard, exactly following the protocol reported by Xu et al.[3]

Text S3. Calculation of Apparent Rate Constants at Different pHs

The proportions of the protonated and deprotonated species were calculated based on their p*K*a values (Table S2). Then, the apparent rate constants were calculated according to the following equations at three pHs (5.5, 7.1, and 7.8), based on the specie-specie rate constants.

Table S2. Oxidants' pKa and speciation

oxidants	p*K*a	species	pH 5.5	pH 7.1	pH 7.8
PFA	7.3[4]	α_{HCOOOH}	98.44%	61.31%	24.02%
PAA	8.2[5]	$\alpha_{CH3COOOH}$	99.80%	92.64%	71.52%
HOBr	8.8[6]	α_{HOBr}	99.95%	98.04%	90.91%
		α_{OBr-}	0.05%	2.16%	9.09%
HOI	10.4[7]	α_{HOI}	99.99%	99.95%	99.75%
		α_{OI-}	0.001%	0.05%	0.25%
H_2O_2	11.6[8]	α_{HO2-}	7.94×10^{-7}	3.16×10^{-5}	1.58×10^{-4}

$$k_{\mathrm{app,HOBr,H_2O_2}} = k_{\mathrm{HOBr,HO_2^-}} \times \alpha_{\mathrm{HOBr}} \times \alpha_{\mathrm{HO_2^-}} \quad \text{(S6)}$$

$$k_{\mathrm{app,HOI,H_2O_2}} = k_{\mathrm{HOI,HO_2^-}} \times \alpha_{\mathrm{HOI}} \times \alpha_{\mathrm{HO_2^-}} \quad \text{(S7)}$$

$$k_{\mathrm{app,PAA,X}} = k_{\mathrm{CH3COOOH,X}} \times \alpha_{\mathrm{CH3COOOH}},\ \mathrm{X = Br^- \ or\ I^-} \quad \text{(S8)}$$

$$k_{\mathrm{app,HOBr,I^-}} = k_{\mathrm{HOBr,I^-}} \times \alpha_{\mathrm{HOBr}} \quad \text{(S9)}$$

Table S3. Conditions for batch kinetic experiments

Conditions	Measurement	Simulated reaction in Table 2 (main text)
$[PFA]_0$ = 100 µM, $[Cl^-]_0$ = 0-0.8 M	PFA decay	R3
$[PFA]_0$ = 100 µM, $[Br^-]_0$ = 0-4.0 mM	PFA decay	R4
$[PFA]_0$ = 100 µM, $[phenol]_0$ = 4.0 mM, $[I^-]_0$ = 10 µM	iodophenol formation	R5
$[PFA]_0$ = 200 µM, $[HOBr]_0$ = 400 µM	bromate formation	R6
$[PFA]_0$ = 200 µM, $[I^-]_0$ = 20 µM	iodate formation	R7
$[PAA]_0$ = 200 µM, $[HOBr]_0$ = 400 µM	bromate formation	R14
$[PAA]_0$ = 200 µM, $[I^-]_0$ = 20 µM	iodate formation	R15

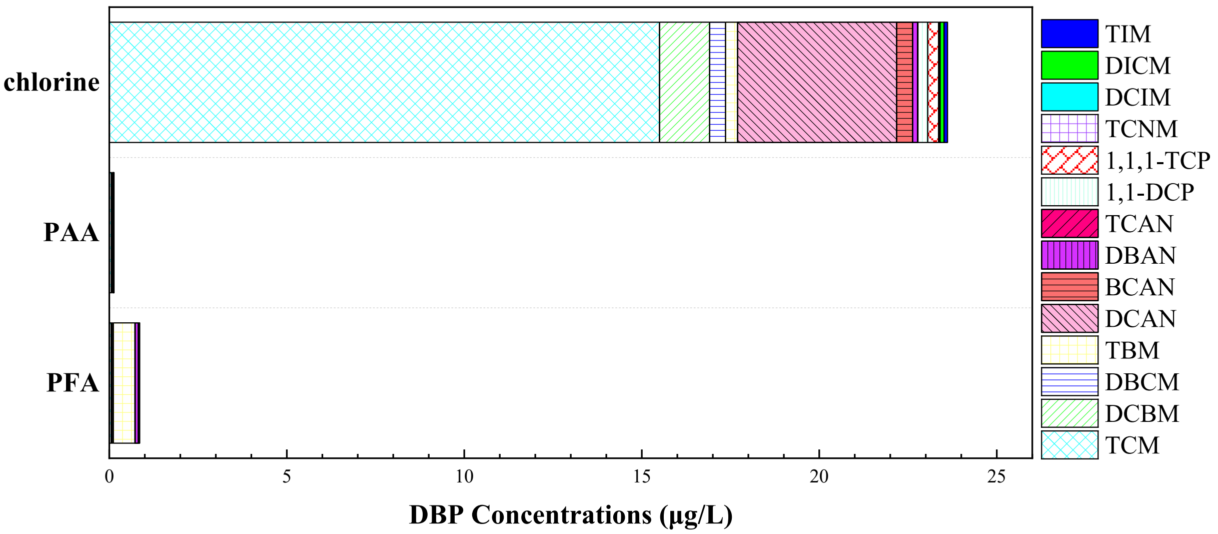

Figure S1. Production of 14 DBPs during disinfection of regular wastewater. Experimental conditions: [phosphate buffer] = 10 mM, $[\text{disinfectant}]_0$ = 100 μM, PAA and PFA both contained 60 μM H_2O_2, initial pH = 7.1, reaction time = 30 min, temperature = 23 ± 2 °C.

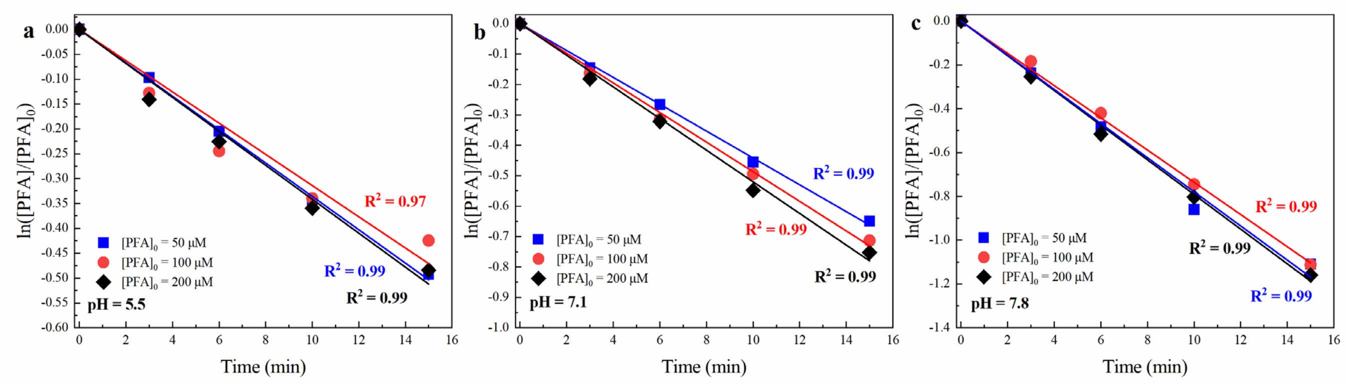

Figure S2. Effect of initial concentration of PFA on PFA decay at pH 5.5 (a), 7.1 (b), and 7.8 (c). Experimental conditions: [phosphate buffer] = 10 mM, temperature = 23 ± 2 °C. Error bars represent standard deviation between parallel experiments. The solid lines represent linear regression modeling. This figure also appears as the supporting information in another submitted manuscript.

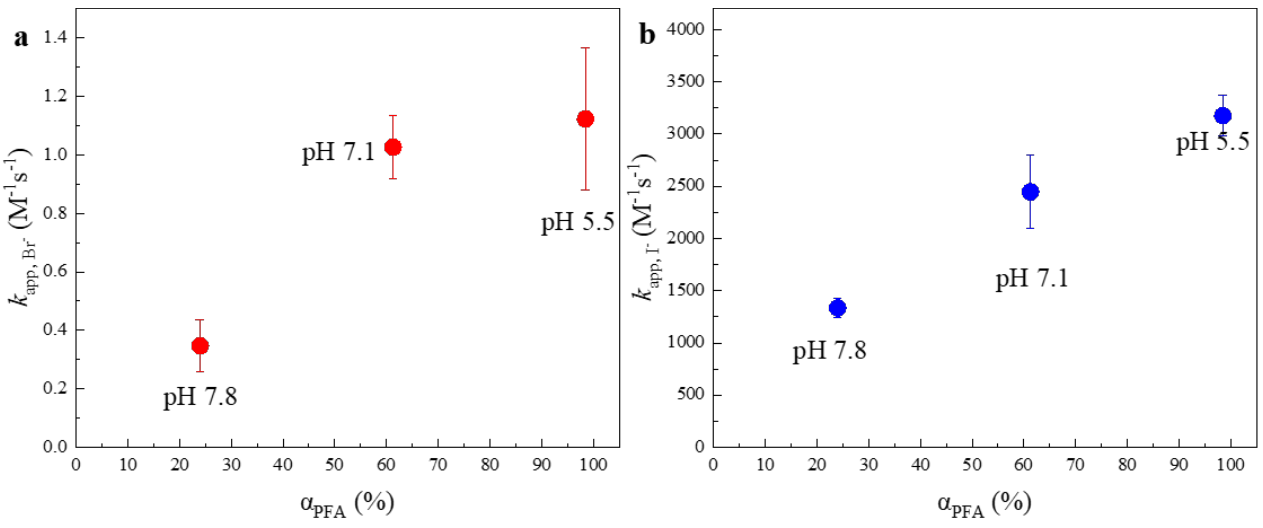

Figure S3. Second-order rate constants between PFA with bromide (a) and iodide (b), versus the proportion of protonated PFA. Experimental conditions: [phosphate buffer] = 10 mM, temperature = 23 ± 2 °C. Error bars represent standard deviation between parallel experiments.

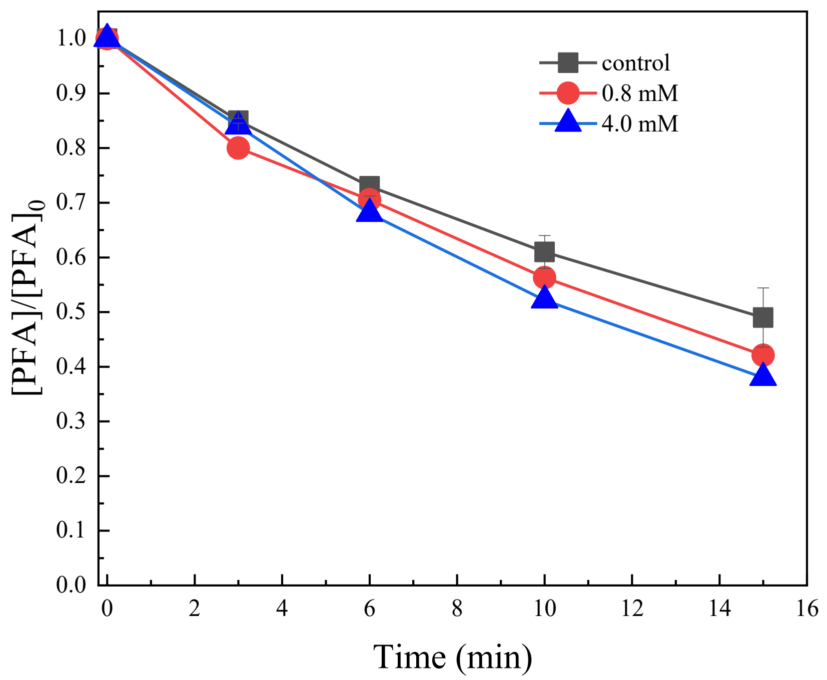

Figure S4. Decay of performic acid in the presence of phenol. Experimental conditions: pH = 7.1, [phosphate buffer] = 10 mM, $[PFA]_0$ = 100 µM, $[phenol]_0$ = 0-4.0 mM, temperature = 23 ± 2 °C, error bars represent standard deviation between duplicate experiments.

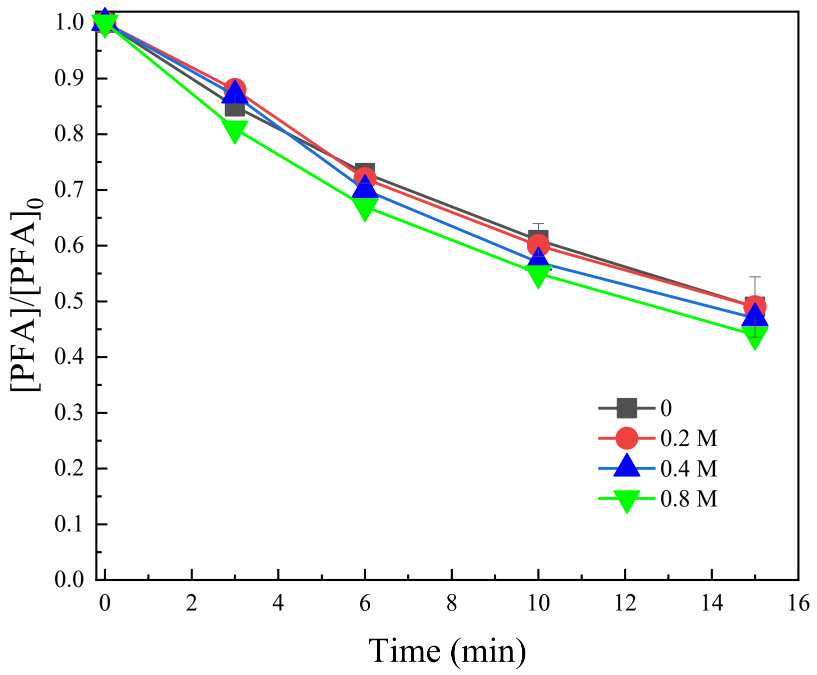

Figure S5. Decay of performic acid in the presence of chloride. Experimental conditions: pH = 7.1, [phosphate buffer] = 10 mM, $[PFA]_0$ = 100 μM, $[Cl^-]_0$ = 0-0.8 M, temperature = 23 ± 2 °C, error bars represent standard deviation between duplicate experiments.

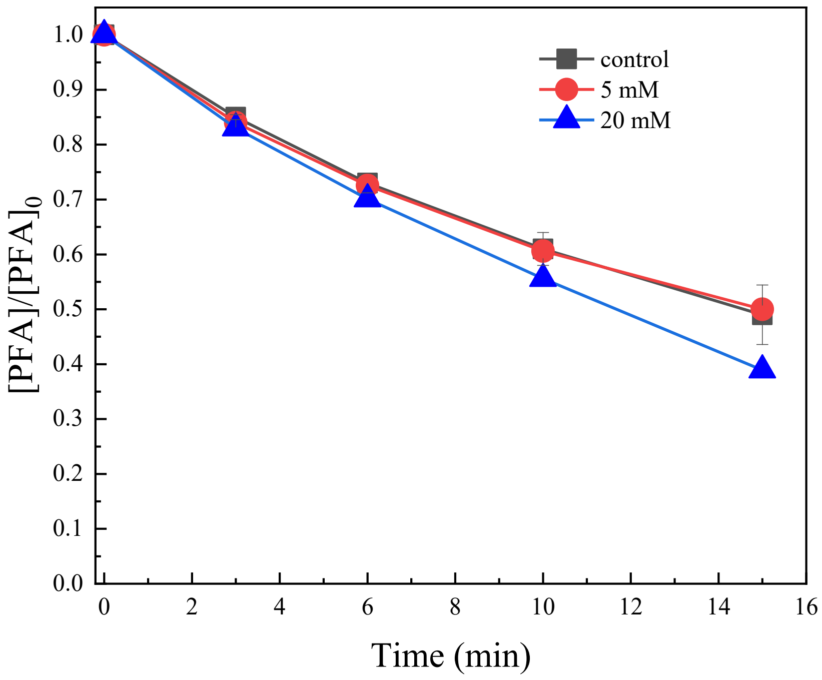

Figure S6. Decay of performic acid in the presence of ammonia. Experimental conditions: pH = 7.1, [phosphate buffer] = 10 mM, $[PFA]_0$ = 100 μM, $[NH_4^+l]_0$ = 0-20 mM, temperature = 23 ± 2 °C, error bars represent standard deviation between duplicate experiments.

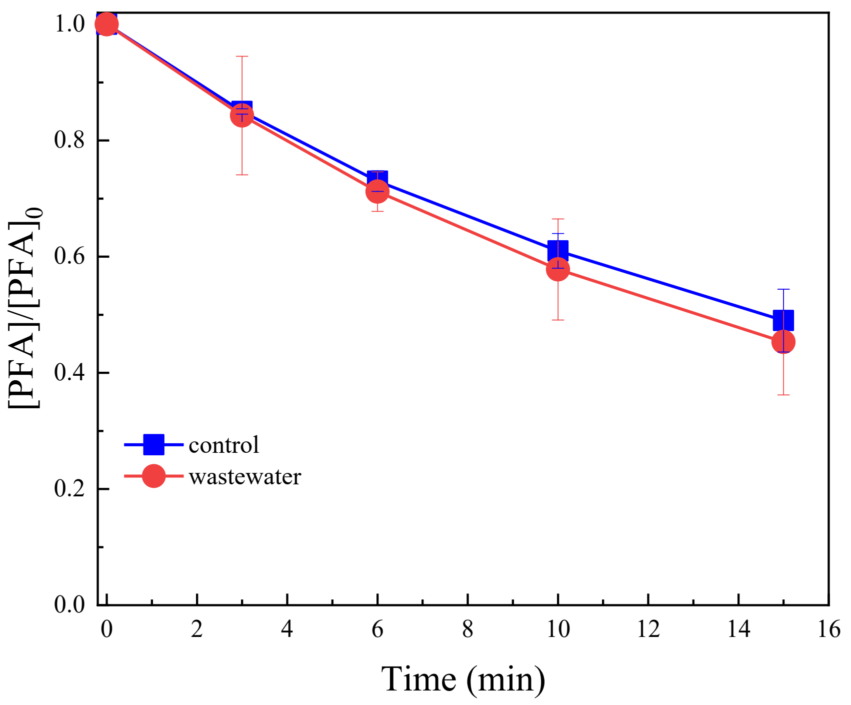

Figure S7. Decay of performic acid in regular wastewater (no extra halides). Experimental conditions: pH = 7.1, [phosphate buffer] = 10 mM, $[PFA]_0$ = 100 μM, temperature = 23 ± 2 °C, error bars represent standard deviation between duplicate experiments.

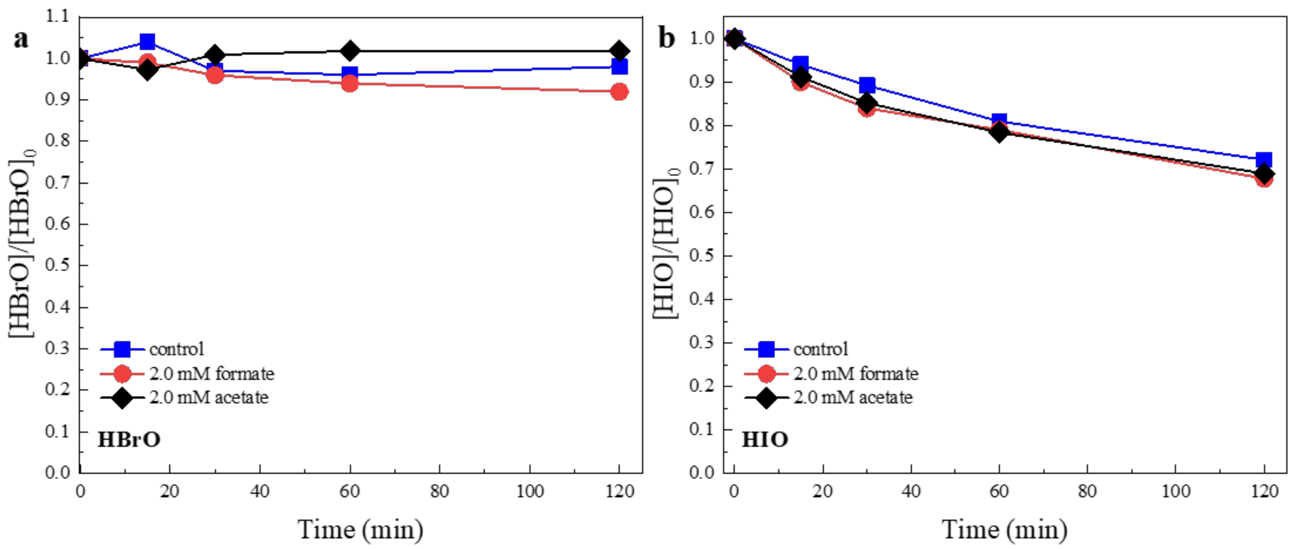

Figure S8. Decay of free bromine (a) and free iodine (b) in the presence of acetate and formate. Experimental conditions: pH = 7.1, [phosphate buffer] = 10 mM, [free bromine]$_0$ = 100 µM, [free iodine]$_0$ = 10 µM, [formate] = [acetate] = 2.0 mM, temperature = 23 ± 2 °C.

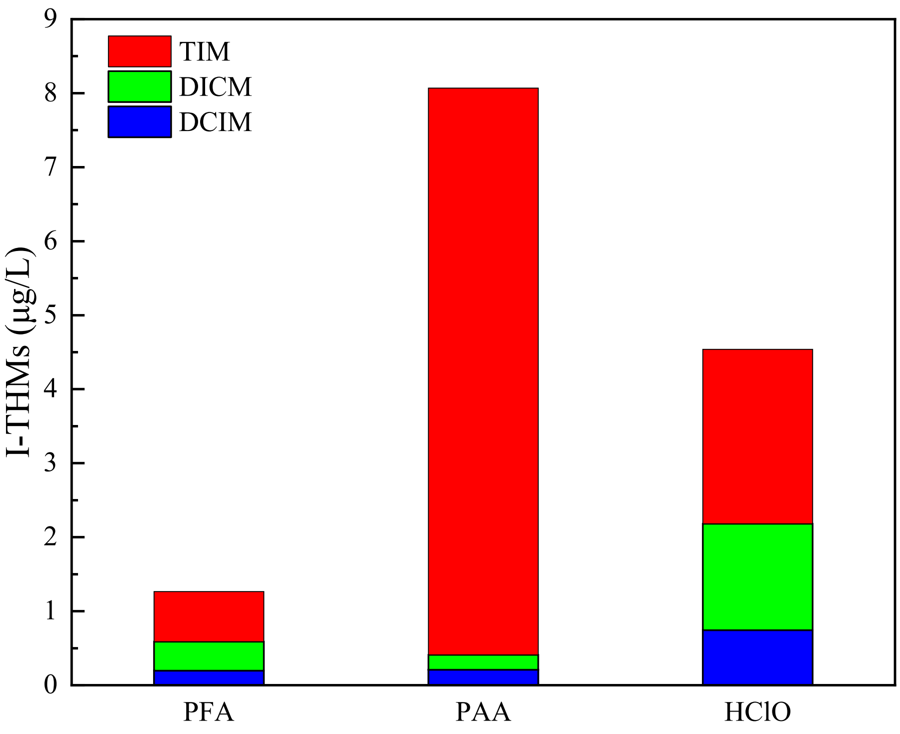

Figure S9. Formation of I-THMs in wastewater spiked with iodide (no extra bromide or chloride). Experimental conditions: [iodide] = 0.3 μM, [phosphate buffer] = 10 mM, $[\text{disinfectant}]_0$ = 100 μM, initial pH = 7.1, reaction time = 24 h, temperature = 23 ± 2 °C. TIM = triiodomethane, DICM = diiodochloromethane, DCIM = dichloroiodomethane.

References

1. Cai, M.; Sun, P.; Zhang, L.; Huang, C. H., UV/Peracetic Acid for Degradation of Pharmaceuticals and Reactive Species Evaluation. *Environ Sci Technol* **2017,** *51*, (24), 14217-14224.

2. Bichsel, Y.; von Gunten, U., Determination of Iodide and Iodate by Ion Chromatography with Postcolumn Reaction and UV/Visible Detection. *Analytical Chemistry* **1999,** *71*, (1), 34-38.

3. Xu, J.; Kralles, Z. T.; Dai, N., Effects of Sunlight on the Trichloronitromethane Formation Potential of Wastewater Effluents: Dependence on Nitrite Concentration. *Environ Sci Technol* **2019,** *53*, (8), 4285-4294.

4. Turco, R.; Tesser, R.; Russo, V.; Cogliano, T.; Di Serio, M.; Santacesaria, E., Epoxidation of Linseed Oil by Performic Acid Produced In Situ. *Industrial & Engineering Chemistry Research* **2021,** *60*, (46), 16607-16618.

5. Kim, J.; Huang, C.-H., Reactivity of Peracetic Acid with Organic Compounds: A Critical Review. *ACS ES&T Water* **2020,** *1*, (1), 15-33.

6. Heeb, M. B.; Criquet, J.; Zimmermann-Steffens, S. G.; von Gunten, U., Oxidative treatment of bromide-containing waters: formation of bromine and its reactions with inorganic and organic compounds--a critical review. *Water Res* **2014,** *48*, 15-42.

7. Shin, J.; von Gunten, U.; Reckhow, D. A.; Allard, S.; Lee, Y., Reactions of Ferrate(VI) with Iodide and Hypoiodous Acid: Kinetics, Pathways, and Implications for the Fate of Iodine during Water Treatment. *Environ Sci Technol* **2018,** *52*, (13), 7458-7467.

8. Shah, A. D.; Liu, Z. Q.; Salhi, E.; Hofer, T.; von Gunten, U., Peracetic acid oxidation of saline waters in the absence and presence of H (2)O (2): secondary oxidant and

disinfection byproduct formation. *Environ Sci Technol* **2015,** *49*, (3), 1698-705.
